# Developmental Heterogeneity in DNA Packaging Patterns Influences T-Cell Activation and Transmigration

**DOI:** 10.1371/journal.pone.0043718

**Published:** 2012-09-05

**Authors:** Soumya Gupta, Nimi Marcel, Shefali Talwar, Megha Garg, Indulaxmi R., Lakshmi R. Perumalsamy, Apurva Sarin, G. V. Shivashankar

**Affiliations:** 1 National Centre for Biological Sciences, Tata Institute for Fundamental Research, Bangalore, Karnataka, India; 2 Mechanobiology Institute and Department of Biological Sciences, National University of Singapore, Singapore; 3 Department of Health Sciences, Manipal University, Manipal, Karnataka, India; 4 Department of Biotechnology, Mysore University, Mysore, Karnataka, India; McGill University Health Center, Canada

## Abstract

Cellular differentiation programs are accompanied by large-scale changes in nuclear organization and gene expression. In this context, accompanying transitions in chromatin assembly that facilitates changes in gene expression and cell behavior in a developmental system are poorly understood. Here, we address this gap and map structural changes in chromatin organization during murine T-cell development, to describe an unusual heterogeneity in chromatin organization and associated functional correlates in T-cell lineage. Confocal imaging of DNA assembly in cells isolated from bone marrow, thymus and spleen reveal the emergence of heterogeneous patterns in DNA organization in mature T-cells following their exit from the thymus. The central DNA pattern dominated in immature precursor cells in the thymus whereas both central and peripheral DNA patterns were observed in naïve and memory cells in circulation. Naïve T-cells with central DNA patterns exhibited higher mechanical pliability in response to compressive loads *in vitro* and transmigration assays *in vivo*, and demonstrated accelerated expression of activation-induced marker CD69. T-cell activation was characterized by marked redistribution of DNA assembly to a central DNA pattern and increased nuclear size. Notably, heterogeneity in DNA patterns recovered in cells induced into quiescence in culture, suggesting an internal regulatory mechanism for chromatin reorganization. Taken together, our results uncover an important component of plasticity in nuclear organization, reflected in chromatin assembly, during T-cell development, differentiation and transmigration.

## Introduction

T-cells in the mammalian immune system circulate in the periphery, exhibiting entry and exit from varied tissues for their function [1]. Tissue transmigration and its subsequent activation is a necessary component of T-cell lineage [2]. These cells are derived from hematopoietic precursors in the bone marrow [3] and their developmental programs must incorporate elements that permit nuclear plasticity for their subsequent differentiation and function [4], [5], [6], [7], [8], [9]. Since the nucleus forms the rigid component of cellular architecture, mechanical pliability to enter tissue requires large-scale deformations of the cell nucleus [10].

Moreover, recent evidences suggest a coupling between gene expression and 3D organization of chromatin [5], [6], [11] - however the underlying principles of this spatio-temporal coupling are poorly understood. Global changes in gene expression characterize T-cell activation [12], which is regulated at multiple levels such as epigenetic modifications on core histone proteins [13], [14], turnover of chromatin binding proteins [15], [16], repositioning of genes and chromosomes [17], [18] and the activity of transcription factors such as Foxp3, NF-κB, GATA family [19], [20], [21]. During T-cell activation, increased activity at the IL2 locus is accompanied by loss of histone proteins such as H3 and H4 [16], whereas the CD69 locus undergoes an exchange with histone variant H3.3 from H3 proteins to facilitate expression [15]. Activity dependent changes in the gene position from nuclear periphery to interior have been noted for β-globin gene during erythroid maturation [17]. In some instances, in addition to gene movements, chromosome positions may also be altered for differential transcriptional outputs [18], [22]. Therefore, cells that transmigrate and are subsequently activated must demonstrate differential nuclear plasticity and chromosome organization to enable this process, although the underlying principles remain to be understood. Here, we map structural transitions in higher order DNA organization within the 3D nuclear architecture (DNA assembly) during the development of T-cells, which offers a unique model system to investigate the coupling between nuclear plasticity and cellular differentiation using a combination of live-cell analysis, immuno-fluorescence and *in vivo* transmigration assays. While circulating T-cells evidenced a heterogeneous DNA assembly, *in vitro* activation resulted in marked redistribution of DNA assembly. In addition, the heterogeneous DNA patterns in circulating T-cells exhibited differential transmigration and activation efficiency.

## Results

**Figure 1:**
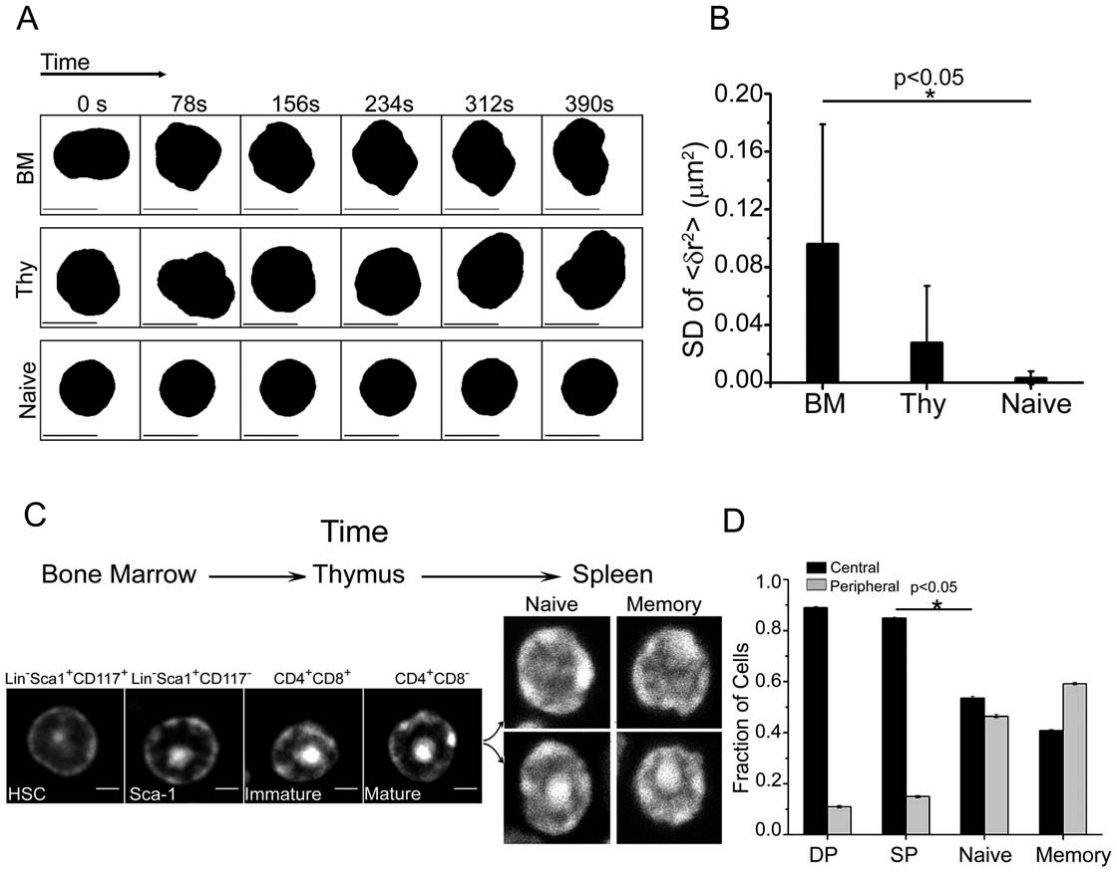
Transitions in nuclear plasticity during T-cell development. a) Representative time series from live-cell imaging of the nuclear boundary in bone marrow, thymocytes and naïve T-cells from H2B-EGFP mice. b) Standard deviation (SD) of mean square fluctuation about the nuclear radius in bone marrow derived cells, thymocytes and naïve T-cells (n = 10 cells each). Error bars are SD. c) Representative images of nuclei stained for DNA pattern with Hoechst 33342 in cells isolated from bone marrow, thymus and spleen. Scale bar 2 µm. d) Quantitative plot scoring for the two different DNA patterns in field images for single positive (SP) and double positive (DP) thymocytes, naïve and memory T-cells (n = 1000 cells each). Error bars are standard error.

To study spatio-temporal transitions in chromatin assembly during T-cell development, cells were isolated from different lymphoid organs of mice including the bone marrow (BM), thymus (Thy) and naïve T-cells from spleen. Time lapse imaging of these cells obtained from H2B-EGFP transgenic mice were used to assess the physical plasticity of nucleus [23], [24], [25]. Time series of mean square fluctuation [<(δr)^2^> = Σ(δr_i_)^2^/N] of the nuclear radius was computed over all angles from the centroid position, using a custom written LabVIEW program. In these experiments, bone marrow cells exhibited large-scale fluctuations in nuclear envelope, whereas thymocytes showed intermediate and naïve T-cells were characterized by highly diminished fluctuations (Figure 1a and b, movies S1, S2, S3). These fluctuations arise due to both nuclear and cytoskeletal dynamics. The structural transitions in nuclear plasticity during T-cell development are consistent with earlier reports [23], [24], [25].

We then visualized DNA using the DNA binding dye Hoechst 33342 in cells isolated from different lymphoid organs of mice. Lineage negative hematopoietic stem cells (HSC) isolated from bone marrow, double-positive CD4^+^CD8^+^ (DP) and single-positive CD4^+^CD8^−^ (SP) thymocytes (Figure S1), and CD4^+^ naïve and memory T-cells showed distinct patterns of condensed DNA distribution (Figure 1c). The distribution of DNA patterns was quantified manually through field images (Figure S2(i)). Independently, this was confirmed with other nucleic acid binding dyes namely propidium iodide (PI) and Sytox green (Figure S2(ii)). Staining patterns of Heterochromatin binding Protein 1 (HP1α) overlapped with that of condensed DNA confirming the latter to be heterochromatin. HSCs have preferential organization of condensed DNA towards the nuclear centre and less in the periphery. This central DNA pattern is also pronounced in DP and SP thymocyte subsets (89% and 85% respectively). However, naïve and memory subsets in circulation were marked by heterogeneity in DNA organization, with only 53% naïve and 40% memory cells presenting the central pattern (Figure 1d). CD8^+^ naïve T-cells also exhibited similar heterogeneity in DNA patterns (Figure S2(iii)). T-cells derived from blood also exhibited heterogeneity in DNA assembly patterns similar to that of splenic naïve T-cells (Figure S2(iv)).

**Figure 2:**
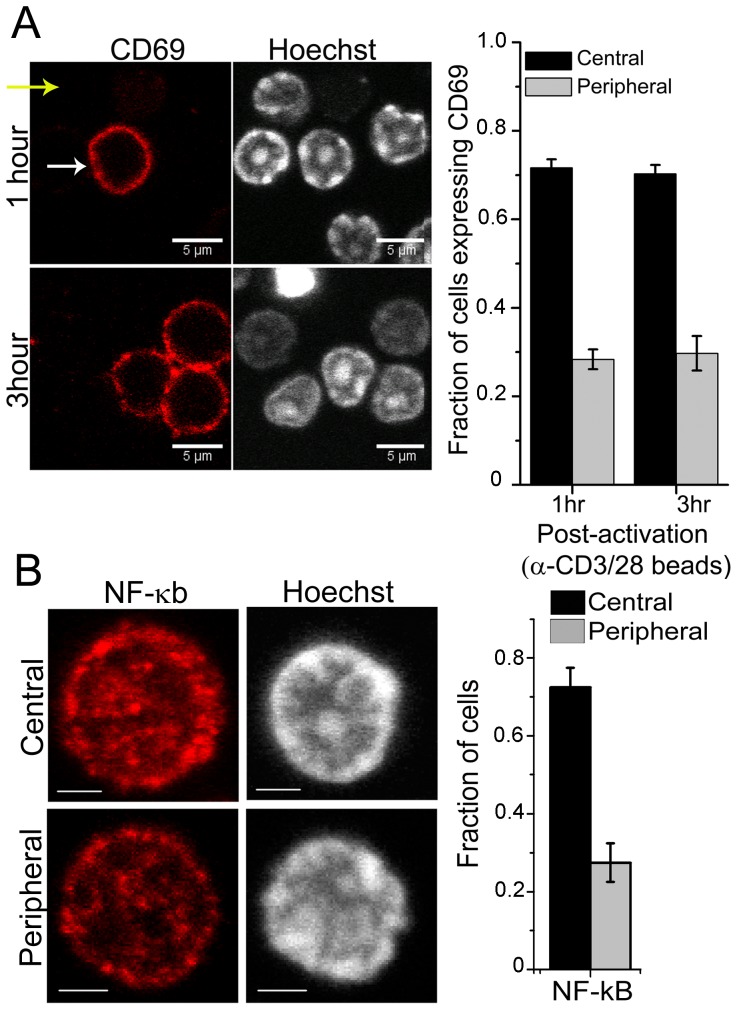
Functional correlations between DNA patterns and transcriptional activity in T-cells. a) Representative images of CD69 staining and DNA and graph showing fraction of activated cells expressing CD69 and having central or peripheral DNA pattern. Error bars are SD. b) Yellow and white arrows point to cells with peripheral and central patterns of DNA, respectively. Naive T-cells were stained for NF-κB, counterstained with Hoechst 33342 and imaged. Representative confocal images from a minimum of 300 cells analyzed. Scale bar 2 µm. The graph shows the fraction of cells with the two different DNA pattern in the three conditions. Data from a minimum of 38 cells are plotted as mean ± SD from two independent experiments. Cells included for analysis were selected as described in text. b).

To test if the heterogeneity in DNA patterns influences early activation and gene expression, naive T-cells were activated *in vitro* with surrogate antigens, αCD3-αCD28 coated beads. 70% of cells with central DNA patterns showed up-regulation of CD69, an early activation gene [26], at both 1 and 3 hours post-activation (Figure 2a). To establish *in vivo* functional significance of heterogeneous DNA patterns, mice were challenged with the superantigen Staphylococcus enterotoxin A (SEA) and the SEA reactive Vβ3^+^ subset of T-cells tracked for early evidence of activation by up-regulation of CD69. Interestingly, the *in vivo* challenge replicated the observation of faster activation of cells with the central pattern of DNA as evident by CD69 expression on these cells (Figure S3). This observation is in concert with the *in vitro* activation data. CD69 expression is regulated via NF-κB [27], hence we tested if cells with central DNA pattern were poised for transcription of CD69. Immuno-fluorescence analysis of cells stained for NF-κB revealed that 15–20% cells stained for levels above full-width at half maximum. Interestingly, this population was enriched for cells with the central pattern of DNA (Figure 2b and Figure S3). Collectively, these experiments suggest a possible correlation between sub-nuclear chromatin organization, NF-κB levels and CD69 expression.

**Figure 3:**
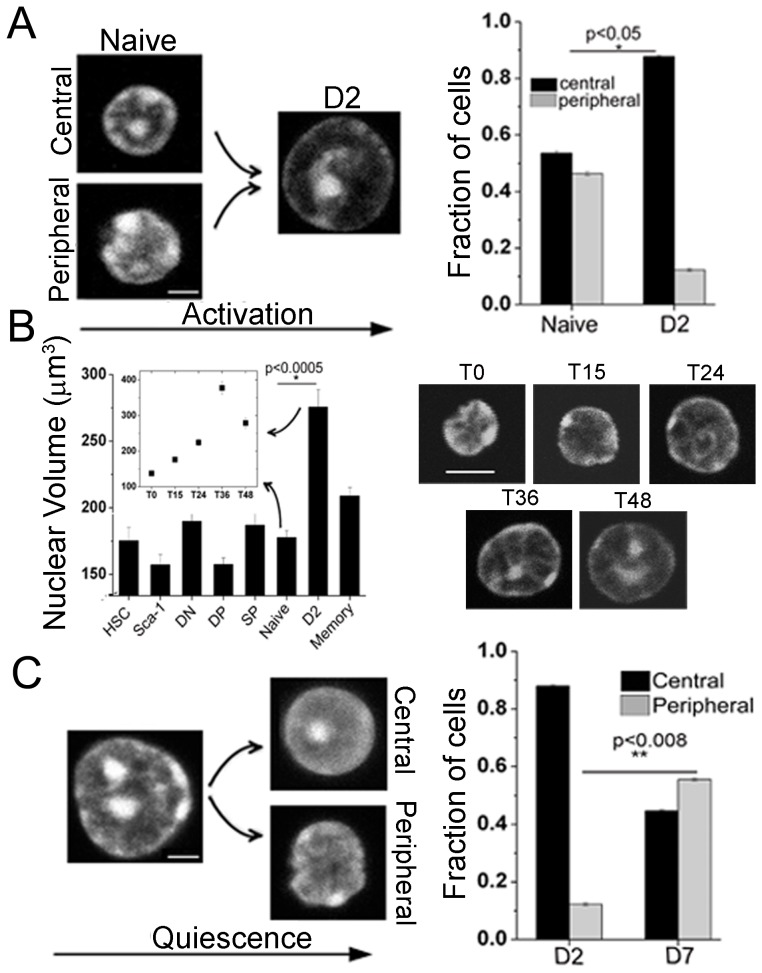
Higher order chromatin reorganization during *in vitro* T-cell activation. a) Representative images of naïve cells and 48 hours post activation (D2) cells stained with Hoechst 33342. Scale bar 2 µm. Quantitative plot scoring for the two different DNA patterns in field images for naïve and activated cells (n = 1000 cells each). b) Nuclear volume (µm^3^) calculated from 3D reconstruction of confocal z-stack images of nuclei (n = 50 cells each). Inset- nuclear volume (µm^3^) for naïve cells getting activated from 0–48 hours. Right hand side shows gradual changes in DNA pattern in naïve T-cells at various time points post-activation. Numbers indicate the hours post-activation of cells. Scale bar 5 µm. c) Representative images of activated (D2) cells and activated cells maintained in culture for 5 more days in IL-7 (D7) stained with Hoechst 33342. Scale bar 2 µm. Quantitative plot scoring for the two different DNA patterns in field images for activated and D7 cells (n = 1000 cells). Error bars indicate SE in all panels.

Since peripheral T-cells present large scale changes in gene expression within 48 hours after encountering antigens [28], therefore we assessed the structural transitions, if any, in DNA patterns during activation. The heterogeneous patterns of DNA in naïve T-cells were markedly reduced upon activation, with 88% activated cells presenting centrally located DNA (Figure 3a). Activation of T-cells was confirmed by staining cells for expression of activation markers- CD69 and CD25 (Figure S4(i)). Activation protocols using either the co-stimulatory molecule CD134, or maleylated ovalbumin as the antigen to activate OT-II transgenic T-cells, also resulted in similar reorganization of DNA (Figure S2(ii)). Figure S4(ii) shows the fraction of cells in various stages of cell-cycle following αCD3-αCD28 activation after 48 hours suggesting that these transitions in DNA patterns were not an outcome of cell cycle distribution. T-cell activation and subsequent quiescence were also marked by changes in nuclear size, which were quantified using a custom written LabVIEW program for 3D reconstruction of confocal z-stacks (Figure 3b). Time-course kinetics experiments revealed concomitant changes in nuclear size and DNA organization during T-cell activation (Figure 3b inset).

The activation of T-cells was accompanied with repositioning of peripheral DNA pattern towards the center and hence we next probed if reorganization of DNA patterns accompanies the induction of quiescence in these cells. For this, activated cells were induced to quiescence by maintaining them in IL7 for five days (D7 cells). These cells exhibited the reemergence of heterogeneity; with 45% of the cells presenting the central DNA pattern in contrast with 88% in activated cells (Figure 3c). In contrast with naïve T-cells, flow sorted SP thymocytes stimulated with αCD3-αCD28 coated beads maintained their central DNA patterns and subsequent induction of quiescence in 85% of cells (Figure S4(iii)). Alterations in DNA patterns were not a consequence of manipulations in cell culture, since patterns of DNA assembly remain unchanged following extended culture of un-stimulated SP thymocytes (85% central) or naïve T-cells (53% central) in IL7 (Figure S4(iii)). These experiments suggest that SP cells when stimulated did not alter their DNA patterns while naïve T-cells when activated presented with a more homogenous DNA pattern. However, when naïve T-cells were maintained in culture without activation, the patterns remained unchanged. In addition, D2 cells maintained in culture until D7, showed the re-emergence of heterogeneity in DNA patterns. The central and peripheral patterns of heterochromatin in cells so rested were similar to those observed in naive and memory cells.

Post-translational modifications play a central role in regulating structural compaction of chromatin and thus genome function [29], [30]. The N-terminal tails of core histone proteins are sites for a variety of modifications including acetylation, phosphorylation and methylation [31]. To test if reorganization of the peripheral DNA and the nuclear volume increase in activated T-cells was correlated with histone modifications, core histone acetylation (H3Ac, H3K9Ac) and methylation (H3K4me2, H3K27me3, H3K9me) were quantified using immuno-fluorescence and flow cytometry (Figure S5(i)). The trend in MFI values that we observe for histone stains were similar to that of the background, suggesting that accessibility of chromatin to primary antibodies were sensitive to stages of development, in agreement with a recent report [32]. This recent report establishes aspects of chromatin reorganization during T-cell differentiation. Importantly, their results highlight the significance of chromatin decompaction during T-cell activation. Figure S5(ii) also reveal differences in chromatin staining by either H3Ac or HP1α antibodies in naïve and activated cells. While activated T-cells were permissive to both antibodies, naïve T-cells stained with only H3Ac, consistent with the chromatin accessibility findings reported recently [32]. Further, peripheral nuclear regions showed an enhanced staining for H3Ac in activated cells (Figure S5(iii)). These results suggest that the spatial distribution of activation and repressive marks on histones correlated with the re-organization of chromatin assembly. Supporting this, addition of Histone Deactylase inhibitor (TSA) during activation inhibits the reorganization of chromatin structure (Figure S5(iii)). The variations that we report in post-translational modifications through T-cell differentiation suggest that changes in nuclear volume during differentiation could also possibly result in increased chromatin accessibility.

Consistent with the fact that condensed chromatin moves towards nuclear centre in activated cells, radial positions of candidate gene active chromosomes moved towards the nuclear periphery (Figure S6(i)). These chromosomes (1, 3, 4 & 17) were chosen as they harbor ∼30% of differentially expressed genes related to T-cell activation obtained from the differential gene expression profile (Figure S6(i), Table S1). Since only a fraction of cells (∼33%) enter cell cycle (Figure S4(ii)) following T-cell activation, we speculate that changes in spatial distribution of histone acetylation, and the cytoskeletal network may also contribute to the observed increase in nuclear volume. Supporting this, the microarray analysis shows that several of the up-regulated genes code for cellular architectural proteins (Figure S6(ii)). Further, we tested if large-scale chromatin reorganization results in rearrangement at the scale of gene clusters. For this, chromosome conformation capture (3C) [33] assay was carried out on a candidate histone gene cluster on genomic DNA isolated from thymocytes, naïve and activated T-cells. The histone cluster was chosen due to its large size. This cluster is present on chromosome 13 in a 2.2 Mb region as five patches of histone genes interspersed with patches of non-histone genes and matrix attachment regions [34] (Figure S7(i–iii)). Figure S7(i) shows the reorganization histone gene cluster in thymic, naïve and activated T-cells. Interestingly, the expression profile of histone H3 gene also changes with development. Real Time PCR experiments revealed that the levels of the total H3 mRNA is 1.5 fold higher in thymocytes compared to CD4^+^ naïve cells and 1.7 fold in activated T cells (Figure S7(iv)). Expression mapping of a candidate transgenic gene, H2B-EGFP, also revealed differences in levels during T-cell development (Figure S7(iv)). These results suggest that, in addition to large-scale chromosome movements, individual gene cluster reorganization impinging on gene expression accompany T-cell development.

**Figure 4:**
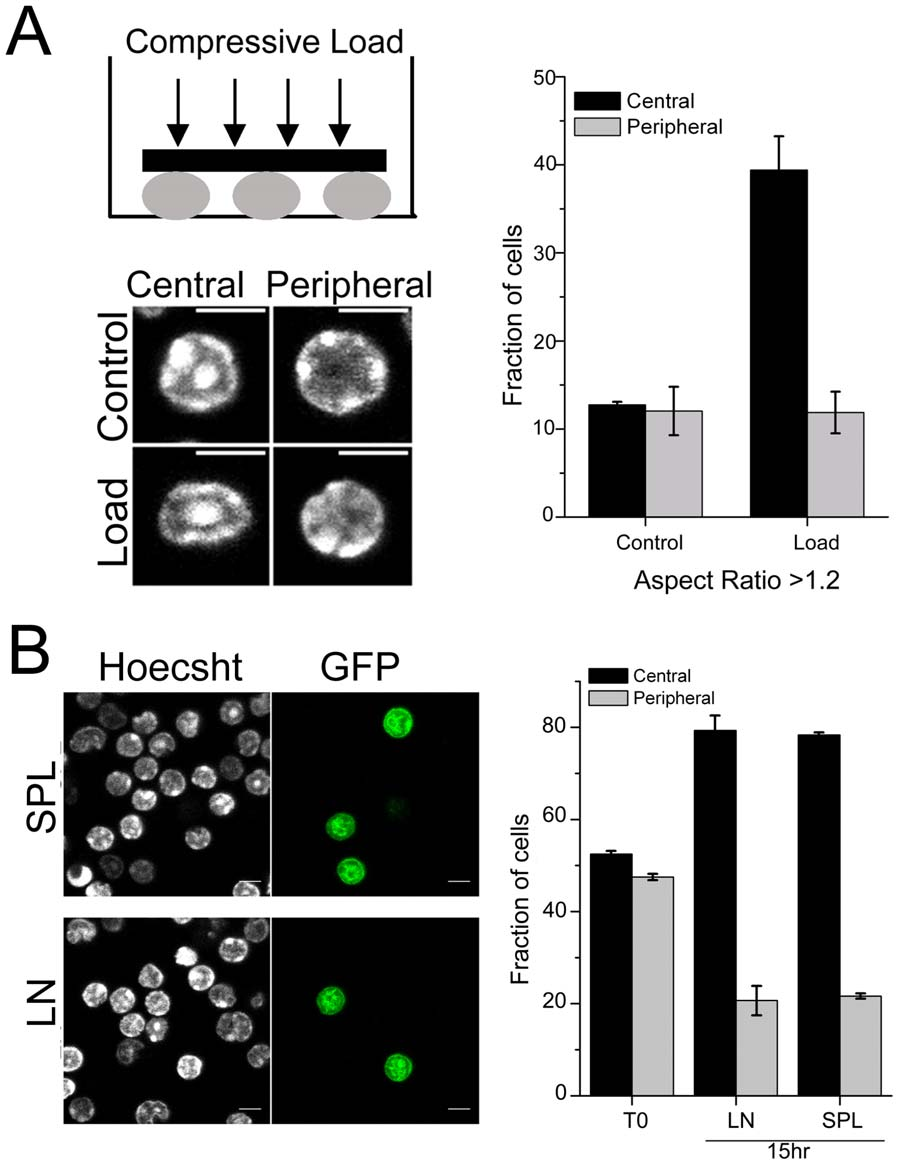
Nuclear deformation response and transmigration properties in T-cells. a) Schematic of experimental design. Representative images of nuclei stained for DNA pattern with Hoechst 33342 in naïve T-cells without (Control) and with compressive load (Load). Scale bar 5 µm. Graph shows the quantitative plot scoring for the two different DNA patterns in field images in naïve T-cells without (Control) and with compressive load (Load) for increase in aspect ratio (n = 500 cells each). Error bars are SD. b) Representative field-views of cells isolated from spleen (SPL) and lymph node (LN) 15 hr post adoptive transfer of GFP^+^ naïve T-cells. Graph shows mean ± S.D of fraction of GFP^+^ cells with the two different DNA patterns in host spleen and lymph node from three independent experiments. n = 150 per experiment.

Cellular transmigration [35] is an essential step during T-cell development where cells in circulation enter and exit from tissue spaces that require mechanical pliability of the cell nucleus. Since our results evidenced heterogeneous DNA patterns in naïve T-cells, we next studied the mechanical responses of the cell nucleus to applied compressive load [36]. Naïve T-cells were subjected to compressive loads (∼1 nN) to test their mechanical pliability as shown in the schematic (Figure 4a). Computing the ratio of the long axis to short axis for individual cells assessed changes in the nuclear morphology. The micrographs in Figure 4a are representative images of control cells and those experiencing load. Interestingly, while there were no differences noted in the aspect ratio of control cells, application of force resulted in increased nuclear deformation in cells with central DNA pattern. The data shows the fraction of cells that demonstrate an aspect ratio of 1.2 or greater in both conditions. While in control cells either DNA patterns were present in equal number, cells upon deformation following the application of load comprised approximately 40% with central and 10% with peripheral DNA patterns (Figure 4a, Figure S8(i–ii)). However, increasing the load to ∼2 nN resulted in comparable nuclear deformations in cells with both DNA patterns (Figure S8(i)), revealing a mechanical bandwidth in nuclear deformations based on DNA packaging. Since nuclear deformation facilitates cellular transmigration, we next assessed if heterogeneity in DNA patterns of naïve T-cells impinged on tissue entry *in vivo*. GFP^+^ naïve T-cells were adoptively transferred into congenic hosts and recovery of GFP^+^ cells tracked in host spleen and lymph nodes at different times points after transfer (Figure 4b and Figure S8(i)) [37], [38]. Consistent with the differential response to compressive load in the *in vitro* assay, cells with central DNA patterns predominated in the GFP^+^ populations that had entered lymph node and spleen. At 15 hours, almost 80% of the cells were enriched in this category (Figure 4b). The micrographs in Figure 4b are representative images of GFP^+^ cells recovered from host tissue. This differential filtering of cellular transmigration to the tissue was maintained even at 72 hours (Figure S8(i)). However, at later time points (7 days after transfer) cells with both DNA patterns were recovered at comparable fractions. Consistent with this data, when we looked at the GFP^+^ cells in blood, cells with peripheral DNA patterns were enriched after 15 hours of adoptive transfer and by 7 days both patterns were observed in comparable fractions in blood (Figure S8(iii)). These results highlight the functional importance of the inherent structural heterogeneity in DNA patterns in circulating naïve T-cells, as these cells are mechanically more pliable and exhibit higher transmigration efficiency.

## Discussion

Taken together our results reveal the functional importance of structural plasticity in chromatin assembly inherent in the processes of cellular differentiation [23], [24], [39]. T-cell development is marked by a reduction in the structural plasticity of the nucleus. We report for the first time, to our knowledge, the emergence of an unusual heterogeneity in higher order chromatin organization and describe two characteristic patterns of DNA assembly in the T-cell lineage. Unexpectedly, mature cells in circulation, exhibit peripheral or central patterns of organization of DNA. Significantly, our experiments reveal that these distinct DNA patterns correlated with differential mechanical responses to applied loads. Thus, cells with central DNA patterns yielded more easily than cells with the peripheral pattern to the same applied force. Importantly, the differential nuclear responses to a mechanical stimulus, correlated with the efficiency of transmigration of cells into peripheral lymphoid organs *in vivo*. The data suggests that cells with the central pattern of DNA are better poised for tissue entry compared to cells with the peripheral DNA pattern. Thus nuclear deformations, determined by DNA packaging patterns, may facilitate tissue entry.

Furthermore, our results show that DNA patterns also correlated with higher levels of NF-κB and early activation markers suggesting that subsets of T-cell population marked by mechanical pliability may be kept transcriptionally poised for subsequent functions in tissues. We also describe global rearrangements in chromatin packaging during the processes of T-cell activation and during progression to quiescence. While naive T-cells have central and peripheral DNA patterns, T-cell activation is marked by redistribution of DNA assembly to the nuclear center. Induction of quiescence revealed an unexpected structural plasticity with the reemergence of heterogeneous DNA patterns as seen in circulating T-cells. Interestingly CD4^+^ SP thymocytes, upon activation and their subsequent quiescence in culture, maintain their central DNA patterns. However the DNA organization remains unchanged, when thymocytes or naïve T cells are kept in culture without activation, suggesting that DNA reorganization is a hallmark of mature T-cell activation and induction of quiescence.

**Figure 5:**
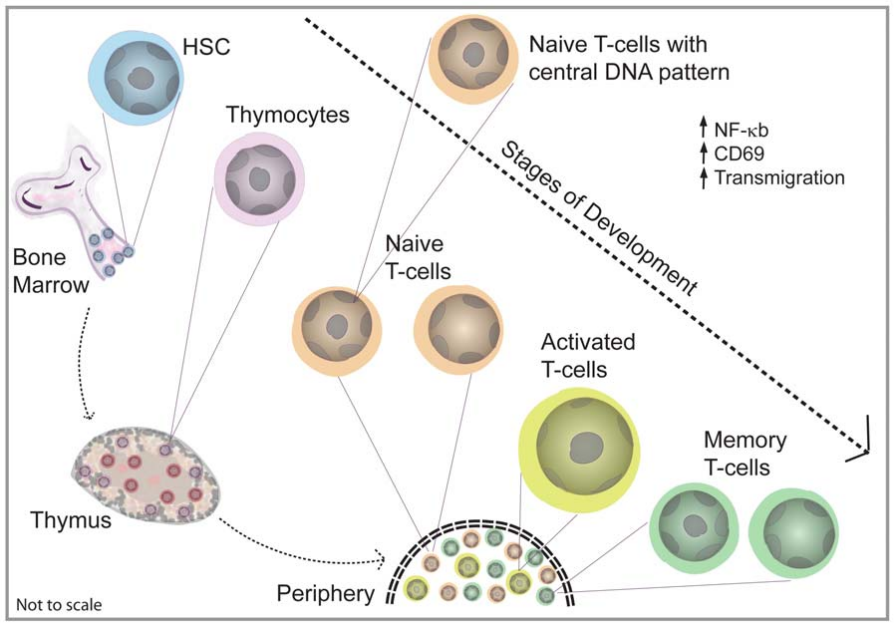
Schematic depicting the various stages in T-cell development and the observed differences in the nuclear organization.

The process of T-cell activation was also coupled with scale-dependent reorganization of higher order chromatin assembly converging on alterations in spatial distributions of histone modifications and gene expression. Significant reduction in peripheral DNA patterns in activated T-cell populations is in consonance with gene active chromosomes residing in the nuclear periphery. The plausible mechanisms for repositioning of heterochromatin can be either movement of chromosomes or by changes in the dynamic association of the nuclear architectural proteins with chromatin. This re-organization in DNA assembly was found to correlate with spatial redistribution of histone acetylation facilitating radial movements of gene active chromosomes and the global gene expression patterns. Figure 5 shows a schematic that summarize our findings. Importantly, circulating cells in blood exhibited heterogeneity in DNA assembly patterns very similar to splenic T-cell population. Further, the *in vivo* activation of T-cells with super-antigens established the functional significance associated with the heterogeneity in DNA assembly patterns (Figure S3).

The emergence and functional transitions in DNA patterns during T-cell development and differentiation appears to be optimized for cellular transmigration and genome function. Since circulating T-cells transmigrate and egress to and from tissue environments, the differences in DNA patterns may provide differential nuclear pliability for these processes. In addition, gene expression is strongly coupled to 3D chromatin structure, and our observations of heterogeneity in DNA packaging may poise subset of cells for distinct genetic outputs.

## Materials and Methods

### Ethics statement

All experiments involving animals were performed with the approval of the Institutional Animal Ethics Committee at NCBS, Bangalore headed by Prof. Mathew with the help of Professors Upinder Bhalla, Sumantra Chatterjee, MM Panicker and R. Sowdhamini. Approval ID for the project is AS-5/1/2008.

### Mice

All experiments used C57/Bl6, transgenic B6.Cg-Tg (Hist1H2BB/EGFP) 1Pa/J or C57BL/6-Tg (TcraTcrb) 425Cbn/J mice (Jackson Laboratories, Bar Harbor, Maine). Mice were bred and maintained in the NCBS animal house facility. Appropriate cell populations from bone marrow, thymus or spleen were isolated using fluorescence-activated cell sorting and magnetic separation. All experiments involving animals were performed with the approval of the Institutional Animal Ethics Committee at NCBS, Bangalore.

### Cell purification and characterization

Hematopoietic Stem cells (HSCs) and CD4^+^ naïve and memory T-cells were isolated using MagCellect kits (R&D Systems) from bone marrow and spleens, respectively, of 6 to 8 week-old mice. Thymocytes were obtained by dissociating thymuses. For some of the experiments, CD4^+^ SP cells were flow sorted after staining with αCD4 and αCD8 antibodies. Whole blood was collected from the heart into PBS containing 30 U/ml heparin. RBCs were removed by centrifugation over histopaque. The cells of interest were identified by staining for characteristic cell surface markers (Figure S1); HSC (Lin^−^Sca-1^+^CD117^+^) and Lin^−^Sca-1^+^CD117^−^ [3], CD4^+^CD8^+^ thymocytes and CD4^+^ naïve (CD62L^hi^CD44^lo^) and CD4^+^ memory (CD62L^lo^CD44^hi^). The antibodies to various cell surface markers were obtained from eBioscience, CA and BD Biosciences.

### T-cell activation

Naïve T-cells were stimulated with beads coated with antibodies to CD3–CD28 (Dynal, Norway) or in some experiments with plate-bound anti-CD3 (1 µg/ml, clone 2C11; R&D Systems) and soluble anti-CD28 (1 µg/ml; BD Biosciences) or anti-CD134 (5 µg/ml, eBioscience). At the end of 48 hrs cultures, beads were removed by magnetic separation, and Day 2 activated T-cells were used for experiments or continued in culture with 10 ng/ml IL-7 (R&D Systems) for 5 days to induce quiescence.

For *in vivo* activation, mice were challenged with 200 ng SEA via tail vein injections. After 5 hours, draining lymph nodes were isolated and cells stained with αVβ3 and αCD69 for 30 minutes on ice; counterstained with Hoechst 33342 followed by analysis using confocal microscopy.

### Force application protocols

Freshly isolated naïve T-cells (10^6^) were stained with Hoechst 33342 to mark DNA and plated on cover-slip dishes in 20 µl culture media and allowed to settle for 5 mins. Force was applied using a metallic O-ring weighing 140 mg stuck to a 13 mm round cover slip deposited gently on the cells. Two magnitudes of force (1 and 2 nN/cell) were applied. Cells were allowed to settle for 15 mins after application of force and imaged using confocal microscope.

### Adoptive transfers

CD4^+^ naïve cells (10×10^6^) isolated from GFP transgenic mice were injected into the tail veins of congenic SJL/Bl6J mice. Cells were recovered from draining lymph nodes and spleen of the host mice at different time points (15 hours, 72 hours and 7 days) post injection. Cells were fixed with 2% PFA and stained with Hoechst to mark DNA and imaged by confocal microscopy. GFP^+^ cells were analyzed for DNA patterns.

### Immuno-staining and flow cytometry

Cells (10^6^) were washed with PBS and fixed with 1% PFA for 20 min, washed twice with PBS, permeabilized with 0.2% NP-40 for 5 mins and washed again twice with PBS. The permeabilized cells were blocked with 10 mg/ml BSA (Sigma Aldrich, MO) in PBS (block) for 1 hr at room temperature (RT). Primary and secondary antibodies were diluted in block and sequentially incubated for 1 hr at RT followed by washes with block. Following antibodies were used: H3Ac, H3K27me_3_, H3K9Ac, H3K9me and H3K4me_2_ (1∶100 dilution, Upstate) HP1α(1∶200 dilution, Upstate), Lamin B1 (1∶1000 dilution, Abcam), NF-κB p65 (1∶50 dilution, Santa Cruz). Secondary antibodies were Alexa488 labelled α-rabbit IgG and α-mouse IgG (1∶500 dilution, Invitogen, CA). Hoechst 33342 (Sigma) was used to visualize DNA. Histone stainings were quantified by flow cytometry in cells in the gate of interest. Flow cytometry data was acquired using a Cyan (Dako) flow cytometer.

### Chromosome painting

For chromosome painting experiments, cells were stuck on PDL coated slides followed by fixation in 4% PFA for 10 minutes. PFA was neutralized with 0.1 M TrisCl and then the cells were washed and permeabilized with 0.5% Triton X-100 for 8 minutes. This was followed by incubation in 20% glycerol for 1 hour and 5 or 6 freeze-thaw cycles in liquid nitrogen. After this, cells were treated with 0.1 N HCl for 10 minutes, washed and equilibrated in 50% Formamide/2× SSC overnight at 4°C. For hybridization cells were denatured in 70% formamide/2× SSC at 85°C for 2 minutes and incubated with the respective chromosome paint (Cambio, Cambridge) for 2–3 days in a moist chamber at 37°C with shaking. At the end of the incubation period, slides were washed thrice each in 50% Formamide/2× SSC at 45°C and 0.1× SSC at 60°C. Cells were counterstained with Hoechst 33342 and mounted with Vectashield (Vector Laboratories, CA), sealed with coverslip and imaged on a Zeiss 510-Meta confocal microscope. 3D centroid positions (*x, y, z*) of the chromosome territories [40] were used to calculate the radial distance (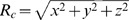) and normalized with respect to nuclear radius to account for the change in nuclear size of activated cells.

### Confocal imaging

A Zeiss (LSM510-Meta) fluorescence microscope was used in our experiments. Imaging was carried out using C-Apochromat 63X/1.4 N.A oil immersion objective with identical acquisition settings. EGFP tagged proteins and Alexa488 were excited with the 488 nm line of an Argon-ion laser (Lassos) and emission collected with a 500–530 nm band pass filter. The fluorophores PE and PECy5 were excited using 543 nm and 633 nm laser line and collected using 560–615 nm band pass and 650LP filters, respectively. 8 bit images of size 512×512 pixels were acquired with optimal pinhole aperture sizes and z-step size of 500 nm.

### Chromosome conformation capture (3C)

Chromosome conformation capture assay was done following the previously reported protocol [33]. Briefly, cells (5×10^4^) were fixed with 4% PFA for 10 mins at RT, followed by neutralization with 0.125 M glycine at 4°C. Cells were washed once with cold PBS and re-suspended and incubated in cold lysis buffer (10 mM Tris, pH 8, 10 mM NaCl, 5 mM MgCl_2_, 0.1 mM EGTA, protease inhibitor cocktail and PMSF) for 10 min at 4°C. Chromatin obtained was digested overnight with *XhoI* enzyme (NEB) at 37°C. Subsequently it was diluted 10 fold and ligated at 16°C overnight. Next proteinase K and RNase treatments were done and finally DNA was precipitated using 3 M NaCl. PCR was carried out with each of the forward primer and the reverse primers of all the downstream sites. The presence of PCR product indicated interaction between two restriction sites. 3C control experiments are shown in Figure S7(ii) which include 1) PCR of genomic DNA without crosslinking, digestion and ligation, 2) PCR of Xho I digested genomic DNA without crosslinking and ligation, 3) PCR of Xho I digested DNA without crosslinking but with ligation and 4) PCR of crosslinked and Xho I digested DNA without ligation. Since the PCR product sizes were very large in our experiments, we based our analysis and model according to position of restriction sites, instead of standard crosslinking frequency analysis [33].

### Microarray analysis

Microarray experiments were carried out by Genotypic (India). Duplicate experiments were done for both naïve and activated T cells. RNA extraction was done using RNeasy Minikit (Qiagen) and RNA concentration and purity were determined using Nanodrop® ND-1000 spectrophotometer (NanoDrop Technologies, Wilmington, DE) and the integrity of RNA verified on an Agilent 2100 Bioanalyzer using the RNA 6000 Nano LabChip (Agilent Technologies, CA). Equal amounts of RNA was labeled using Agilent dye Cy3 CTP and hybridized to *Mus musculus* GeneExpression Array 4X44K (AMADID -014868). The slides were scanned using Agilent Microarray Scanner G2505 version C at 2 µm resolution, and data was extracted from images using Feature Extraction software v 9.5 of Agilent. Mean expression of each gene was calculated by taking an average of all background corrected probes for the same gene. Differentially expressing genes were selected by choosing genes that were more than 10 FWHM (Full Width at Half Maxima) away in the difference histogram between naïve and activated T cells. Genes with the terms ‘cytoskeleton’, ‘actin’, ‘microtubule’ or ‘intermediate filament’, in Gene Ontology were classified as cytoskeletal. Genes located on chromosomes 1,3,4 and 17 were used to plot the chromosome specific differential gene expression in naïve and activated T-cells. The microarray data is MIAME compliant and accessible on GEO website (http://www.ncbi.nlm.nih.gov/geo/; accession number GSE30196).

### Real-time PCR

Total RNA was isolated from thymocytes, CD4^+^ naïve T cells and activated T cells and quantified. 500 ng of RNA from each was used for cDNA synthesis. cDNA synthesis was carried out at 37°C for one hour with random-primed reverse-transcription with M-MuLV Reverse Transcriptase (Bangalore Genei, India). Real time PCR was carried out with the qPCR MasterMix Plus for SYBR Green I No. ROX, Ref RT-SN2X-03+NR (Eurogentec) using ROTOR-GENE RG 3000 (Corbett Research). PCR was carried out in triplicates for two dilutions and average Ct value was calculated for H3. 18S rRNA was used as loading control. Fold change in H3 mRNA was calculated from the Ct values.

### Data analysis

To quantify the fluctuation of the nuclear boundary, time-lapse imaging of H2BEGFP nuclei was done in different cell types and LabVIEW routine was used for edge detection and to find the nuclear radii at different angles in 1° intervals (from 0° to 359°) from its centroid. Mean square changes of the nuclear radius over all the angles, in different time points were used to quantify fluctuation of the nuclear boundary. Change in the nuclear radii (δr_i_) at any time point (t) at angles i (i = 0°, 1°, 2°,..359°) compared to the radii at t = n−1 at the respective angles, were numerically calculated. The mean square fluctuation [<(δr)^2^> = Σ (δr_i_)^2^/N] in the nuclear radii over all angles was plotted at different time points to compute the membrane fluctuation time series. For the analysis of the aspect ratio, cells experiencing compressive load were analyzed for their aspect ratio (ratio of length of the long/short axis). The length of the long and short axis for each cell was obtained as the major and minor axis of the best fitted ellipse using Image J software. Statistical Analysis for all data in bar graphs is presented as mean±SE or SD derived from a minimum of three independent experiments. Statistical significance was calculated using two population student t-test.

## Supporting Information

Figure S1 **Characterization of surface markers for bone marrow and thymic cells.** a) Lineage deprived bone marrow cells were stained for HSC surface markers- Sca-1 and CD117. Representative flow plots showing the expression of these markers are shown. Sca-1a and Sca-1b populations were defined based on slightly different expression of Sca-1. Red- unstained, green- HSC, blue- Sca-1a, pink- Sca-1b. b) MFI averaged from three experiments for the surface markers as specified is plotted. The levels of Sca-1 and CD117 are significantly different in these populations. SE is plotted. c) Representative flow plots showing the expression of surface markers CD4 and CD8 in thymocytes. Red- unstained, green- DP thymocytes, blue- CD4 SP thymocytes. d) MFI averaged from three experiments for the surface markers as specified. Expression of CD4 is similar in DP and CD4 SP thymocytes, but the expression of CD8 is significantly reduced on CD4 SP thymocytes. SE is plotted.
(PDF)

Figure S2 **Confirmation of heterochromatin patterns in T-cells by labelling various markers.** (**i**). Representative field images of nuclei in thymocytes, CD4 naïve and memory cells, and activated T cells, showing the two different condensed DNA patterns observed during different stages of T cell differentiation by Hoechst staining. Central plane of the z-stack is shown. Central or peripheral pattern of DNA were counted from several such images. Scale bar 5 µm. (**ii**). a) Confirmation of DNA pattern in T cells was done by staining the cells with different nucleic binding dyes. Activated cells were treated with RNase A for 30 minutes, then stained with propidium iodide or sytox green. These dyes show co-localization with DNA stained with Hoechst. b) DNA that is stained brightly with Hoechst 33342 is also positive for HP1α, a heterochromatin binding protein. c) Naïve T cells were activated with αCD3-αCD134, a TNFR family co-stimulator molecule, or splenocytes from OT-II TCR transgenic mice were activated with maleylated ovalbumin for three days. T-cells were labelled by staining with αThy1.2, and the DNA imaged. The pattern of DNA organization is similar, irrespective of the kind of stimulation given to cells. Scale bar 2 µm. (**iii**). a) Plot indicating the two types of DNA patterns observed in CD8^+^ naïve T-cells (n = 230 cells each). (**iv**). a) Analysis of heterochromatin pattern in T-cells from blood: Representative images of nuclei stained for DNA pattern with Hoechst 33342 in cells isolated from blood which are positive for surface markers: CD4 (red) and CD62L (green). Scale bar 5 µm. b) Quantitative plot scoring for the two different DNA patterns in field images for cells isolated from blood which are stained positive for CD4 and CD62L. For comparison, naïve cell data from spleen cells is also shown. Error bars are standard error.
(PDF)

Figure S3 **Heterogeneity in DNA patterns influences early activation and gene expression.** a) Representative images of nuclei stained for DNA pattern with Hoechst 33342 (blue) in cells isolated from lymph node of mice challenged with SEA for 5 hrs, which are positive for Vβ3(green) and CD69(red). Scale bar 5 µm. Quantitative plot scoring for the two different DNA patterns in field images for Vβ3^+^ T cells and stained positive or negative for CD69 (n = 152). Error bars are standard deviation. b) Naïve T-cells were stained for NF-κB, counterstained with Hoechst and imaged. Representative confocal field views are shown. Scale bar 5 µm. The plot on right side highlights the cells that were above full width at half maxima. Only those cells were included in analysis of DNA pattern.
(PDF)

Figure S4 **Characterization and analysis of heterochromatin patterns in activated T-cells.** (**i**). Naïve T-cells were activated for 48 hours with αCD3-αCD28 antibody coated eads in absence (D2) or presence of TSA (TSA treated D2 cells). Cells were then stained with αCD69 or αCD25 to confirm activation with the antibody coated beads. Histone deacetylase inhibitor TSA was used as an additional control to assess the role of chromatin perturbation and T-cell activation. (**ii**). Cell cycle analysis for day 2 activated cells. G0–G1, S and G2 phase have 56.9%, 18.49% and 14.7% cells, respectively. Less than 50% of cells have entered cell cycle. (**iii**). a) Representative images and quantitative plot scoring for the two different DNA patterns in field images for single positive (SP) resting, activated and D7 thymocytes (n = 100 cells). b) Representative images of naïve and SP thymocytes maintained in IL-7 for 4 days and the plot indicating the differences in DNA patterns (n = 50 cells each).
(PDF)

Figure S5 **Histone modifications changes during T-cell development.** (**i**). MFI data and representative flow plots showing the histogram of H3Ac, H3K4me2, H3K9Ac, H3K9me, H3K27me3 and background staining (inset) in T–cell subsets from bone marrow (BM), thymus, and spleen. The MFI plots for the various histone antibodies were calculated by subtracting the background control values obtained in the respective population. The levels of these modifications (including background) change during differentiation, being higher in bone marrow cells but lower in thymic cells and increasing again during T-cell activation. (**ii**). Collage of H3Ac staining in a) naïve T-cells b) activated cells and c) rabbit secondary antibody control images. Scale bar 5 µm. Collage of images showing HP1α staining in d) naïve cells and e) activated cells. Scale bar 2 µm. (**iii**). a) H3Ac staining (green) in naïve and D2 cells, DNA counterstained with Hoechst (red) showing the spatial reorganization of H3Ac. b) Representative DNA images of CD4^+^ naïve T-cells activated for 48 hours with αCD3-αCD28 antibody in presence of 10 nM TSA. Scale bar 2 µm. c) Quantitative plot scoring for the two different DNA patterns in field images of naïve and TSA treated (5 nM, 10 nM) or control activated T cells (T48TSA0) (n = 1000 cells each).
(PDF)

Figure S6 **Chromosomal positions and gene expression analysis in naïve and activated T-cells.** (**i**). a) Normalized average distance of centroid of chromosomes from the nuclear centroid (n = 70). Inset- z-projection of DNA (blue) and chromosome paint for chromosome pair 1 (green) and 4 (red). Upon activation, chromosome 1 repositioned towards the nuclear periphery (0.86) as compared to the naïve cells where it was present at 0.76 relative to the nuclear centroid. Chromosomes 3, 4 and 17 also repositioned to more peripheral positions during T-cell activation. Scale bar 2 µm. b) Colour graph representing expression of genes on four candidate chromosomes in naïve and activated cells as observed by microarray data. Darker shades represent lower intensity; lighter shades represent higher intensity. (**ii**). a) Cytoskeleton is an important component in maintaining cell shape and size, and T-cell activation is accompanied by an increase in cell and nuclear size. Differential expression of cytoskeleton related genes in naïve and activated (D2) cells as analyzed by microarray. Higher expression of most cytoskeletal genes by activated cells probably explains the higher nuclear volume of those cells. Gene names are given on the left side. Cooler colors represent lower expression value; warmer colors represent higher expression value. b) Microtubules were stained by α-tubulin antibody and z-stacks were acquired. Shown in the image are z-projections of microtubule staining in naïve and activated T cells. Scale bar 2 µm. Staining for lamin B1, major component of nucleoskeleton, appears similar in naïve and activated cells, however, it appears indeted in naïve cells but smooth and continuous in activated cells. Scale bar 5 µm.
(PDF)

Figure S7 **3C analysis of histone gene cluster during T-cell development.** (**i**). A) Map of histone cluster I on chromosome 13. The rectangles show the position and size of each patch of histone genes in the cluster. The down arrows indicate the position of *Xho I* sites selected. The short horizontal arrows show the position of the primers used. The thick red arrows show the positions of the H3 genes within the cluster. The circles show the sites considered in the schematic of the association of the H3 genes. B) PCR for 3C for the histone gene cluster in mouse cells with different primer combinations, T: thymocyte, N: Naïve and D: D2 activated T cells: Lanes 1,18: 1 kb DNA Ladder; 2: control 1 (without crosslinking, digestion and ligation); lane 3: control 2 (without crosslinking and ligation); lane 4: control 3 (without crosslinking); lane 5: control 4 (without ligation); Lane 6, 7, 8: Primers 1 and 5; lane 9, 10, 11: Primers 2 and 5; lane 12, 13, 14: Primers 2 and 17; lane 15,16, 17: Primers 1 and 3. C) Lane 1, 15: 1 kb ladder; lane 2–5: controls; lane 6,7,8: primers 5 and 6; lane 9,10,11: primers: 5 and 9; lane 12,13,14: primers 5 and 17. D) Cartoon depicting the associations of encircled regions in schematic in panel A in thymocytes, CD4^+^ naïve and activated T cell. (**ii**). 3C control experiments: with different primer combinations. A: 1–16: −crosslink, −digesion, −ligation, lane 17: 1 kb ladder. B: 1–7: crosslink, −digesion, −ligation; 8, 18: marker, 9–17: −crosslink, +Xho I, −ligase. C: 1–12: −crosslink, +Xho I, −ligase; 13–16: −crosslink, +Xho I+ligase, 17: marker. D: 1–17: −crosslink, +Xho I+ligase, 18 marker. E: 1–17: +crosslink, +digestion, −ligation, 18; marker. F: 1–8: +crosslink, +digestion, −ligation, 9; marker. (**iii**). 3C analysis of thymocyte cells shown in a–d. a) lanes 1–10: primers 1F and all the reverse primers; 12–18: primers 5F and all the reverse primers. b) 1–9: primers 2F and all reverse primers;11–18: primers 3F and all reverse primers. c)1–6: primers 6F and all reverses; 8–12: primers 9F and all reverses; 14–16: primers 14F and all reverse primers. d)1–4: primers 12F and all reverses; 6–7: primers 17F and all reverses;9: primers 20F and 20R. 3C analysis of naïve cells shown in e–h. e) lanes 1–10: primers 1F and all the reverse primers; 12–18: primers 5F and all the reverse primers; f)1–9: primers 2F and all reverse primers;11–18: primers 3F and all reverse primers. g)1–6: primers 6F and all reverse primers; 8–12: primers 9F and all reverse primers; 14–16: primers 14F and all reverse primers. h) 1–4: primers 12F and all reverse primers; 6–7: primers 17F and all reverse primers; 9: primers 20F and 20R. 3C analysis of activated T cells shown in i–l. i) lanes 1–10: primers 1F and all the reverse primers; 12–18: primers 5F and all the reverse primers; j)1–9: primers 2F and all reverse primers;11–18: primers 3F and all reverse primers. k)1–6: primers 6F and all reverse primers; 8–12: primers 9F and all reverse primers; 14–16: primers 14F and all reverse primers. l) 1–4: primers 12F and all reverse primers; 6–7: primers 17F and all reverse primers; 9: primers 20F and 20R. (**iv**). a) The expression of H3 normalized to that of 18S is plotted. b) Mean fluorescence intensity plots of H2B EGFP are shown for the different cells (N = 3). Representative images of the Sca-1^+^ and activated T cells for the H2B EGFP are shown in the inset. Scale bar 2 µm.
(PDF)

Figure S8 **Implications of heterogeneity in DNA patterns on naive T-cells functions.** (**i**). a) Representative field-view images of naïve T-cells without (Control) and with different compressive loads (Load1 and Load 2). b) Quantitative plot scoring for the two different DNA patterns in field images in naïve T-cells without (Control) and with higher compressive load of 2 nN (Load2) for increase in aspect ratio (n = 500 cells each). c) Quantitative graph showing the recovery of GFP^+^ cells in lymph node and spleen at later time points (72 hr and 7days post adoptive transfer) and analyzed for DNA patterns. (**ii**). Histogram plot showing the nuclear aspect ratio for cells with central or peripheral pattern of condensed DNA, without (Control) and with compressive load (Load). (**iii**). Fraction of GFP+ naïve T-cells in blood with central and peripheral DNA patterns after 15 hr and 7 days of adoptive transfer into wild type congenic mice. Mean+ SD is plotted.
(PDF)

Movie S1 **Time lapse imaging of H2B-EGFP labeled nucleus in cells from bone marrow to depict the nuclear envelope fluctuations.**
(AVI)

Movie S2 **Time lapse imaging of H2B-EGFP labeled nucleus in cells from thymus to depict the nuclear envelope fluctuations.**
(AVI)

Movie S3 **Time lapse imaging of H2B-EGFP labeled nucleus in naïve T-cells to depict the nuclear envelope fluctuations.** In all movies, the images have been thresholded and converted to binary format.
(AVI)

Table S1 **Table with list of genes located on chromosomes 1,3,4, and 17 and which show differential activity in naïve and activated T-cells.**
(PDF)

## References

[1] Luster AD, Alon R, von Andrian UH (2005) Immune cell migration in inflammation: present and future therapeutic targets. Nat Immunol 6: 1182–1190. 16369557 10.1038/ni1275

[2] Imhof BA, Aurrand-Lions M (2004) Adhesion mechanisms regulating the migration of monocytes. Nat Rev Immunol 4: 432–444. 15173832 10.1038/nri1375

[3] Spangrude GJ, Heimfeld S, Weissman IL (1988) Purification and characterization of mouse hematopoietic stem cells. Science 241: 58–62. 2898810 10.1126/science.2898810

[4] Krangel MS (2007) T cell development: better living through chromatin. Nature immunology 8: 687–694. 17579647 10.1038/ni1484

[5] Lanctot C, Cheutin T, Cremer M, Cavalli G, Cremer T (2007) Dynamic genome architecture in the nuclear space: regulation of gene expression in three dimensions. Nature reviews Genetics 8: 104–115. 10.1038/nrg2041 17230197

[6] Misteli T (2007) Beyond the sequence: cellular organization of genome function. Cell 128: 787–800. 17320514 10.1016/j.cell.2007.01.028

[7] Meshorer E, Misteli T (2006) Chromatin in pluripotent embryonic stem cells and differentiation. Nature reviews Molecular cell biology 7: 540–546. 16723974 10.1038/nrm1938

[8] Kosak ST, Groudine M (2004) Form follows function: The genomic organization of cellular differentiation. Genes & development 18: 1371–1384. 15198979 10.1101/gad.1209304

[9] Fraser P, Bickmore W (2007) Nuclear organization of the genome and the potential for gene regulation. Nature 447: 413–417. 17522674 10.1038/nature05916

[10] Shivashankar GV (2010) Nuclear mechanics and genome regulation. Methods in cell biology 98: xiii. 20816227 10.1016/S0091-679X(10)98019-9

[11] Hubner MR, Spector DL (2010) Chromatin dynamics. Annual review of biophysics 39: 471–489. 10.1146/annurev.biophys.093008.131348 PMC2894465 20462379

[12] Wilson CB, Merkenschlager M (2006) Chromatin structure and gene regulation in T cell development and function. Curr Opin Immunol 18: 143–151. 16472999 10.1016/j.coi.2006.01.013 PMC1820769

[13] Northrop JK, Thomas RM, Wells AD, Shen H (2006) Epigenetic remodeling of the IL-2 and IFN-gamma loci in memory CD8 T cells is influenced by CD4 T cells. J Immunol 177: 1062–1069. 16818762 10.4049/jimmunol.177.2.1062

[14] Kersh EN, Fitzpatrick DR, Murali-Krishna K, Shires J, Speck SH, et al (2006) Rapid demethylation of the IFN-gamma gene occurs in memory but not naive CD8 T cells. J Immunol 176: 4083–4093. 16547244 10.4049/jimmunol.176.7.4083

[15] Sutcliffe EL, Parish IA, He YQ, Juelich T, Tierney ML, et al (2009) Dynamic histone variant exchange accompanies gene induction in T cells. Mol Cell Biol 29: 1972–1986. 19158270 10.1128/MCB.01590-08 PMC2655607

[16] Chen X, Wang J, Woltring D, Gerondakis S, Shannon MF (2005) Histone dynamics on the interleukin-2 gene in response to T-cell activation. Mol Cell Biol 25: 3209–3219. 15798206 10.1128/MCB.25.8.3209-3219.2005 PMC1069623

[17] Ragoczy T, Bender MA, Telling A, Byron R, Groudine M (2006) The locus control region is required for association of the murine beta-globin locus with engaged transcription factories during erythroid maturation. Genes Dev 20: 1447–1457. 16705039 10.1101/gad.1419506 PMC1475758

[18] Kim SH, McQueen PG, Lichtman MK, Shevach EM, Parada LA, et al (2004) Spatial genome organization during T-cell differentiation. Cytogenetic and genome research 105: 292–301. 15237218 10.1159/000078201

[19] Lee KY, D'Acquisto F, Hayden MS, Shim JH, Ghosh S (2005) PDK1 nucleates T cell receptor-induced signaling complex for NF-kappaB activation. Science 308: 114–118. 15802604 10.1126/science.1107107

[20] Long M, Park SG, Strickland I, Hayden MS, Ghosh S (2009) Nuclear factor-kappaB modulates regulatory T cell development by directly regulating expression of Foxp3 transcription factor. Immunity 31: 921–931. 20064449 10.1016/j.immuni.2009.09.022

[21] Ranganath S, Murphy KM (2001) Structure and specificity of GATA proteins in Th2 development. Mol Cell Biol 21: 2716–2725. 11283251 10.1128/MCB.21.8.2716-2725.2001 PMC86902

[22] Mehta IS, Amira M, Harvey AJ, Bridger JM (2010) Rapid chromosome territory relocation by nuclear motor activity in response to serum removal in primary human fibroblasts. Genome Biol 11: R5. 20070886 10.1186/gb-2010-11-1-r5 PMC2847717

[23] Bhattacharya D, Talwar S, Mazumder A, Shivashankar GV (2009) Spatio-temporal plasticity in chromatin organization in mouse cell differentiation and during Drosophila embryogenesis. Biophys J 96: 3832–3839. 19413989 10.1016/j.bpj.2008.11.075 PMC3297759

[24] Pajerowski JD, Dahl KN, Zhong FL, Sammak PJ, Discher DE (2007) Physical plasticity of the nucleus in stem cell differentiation. Proc Natl Acad Sci U S A 104: 15619–15624. 17893336 10.1073/pnas.0702576104 PMC2000408

[25] Meshorer E, Yellajoshula D, George E, Scambler PJ, Brown DT, et al (2006) Hyperdynamic plasticity of chromatin proteins in pluripotent embryonic stem cells. Dev Cell 10: 105–116. 16399082 10.1016/j.devcel.2005.10.017 PMC1868458

[26] Sancho D, Gomez M, Sanchez-Madrid F (2005) CD69 is an immunoregulatory molecule induced following activation. Trends in immunology 26: 136–140. 15745855 10.1016/j.it.2004.12.006

[27] Blazquez MV, Macho A, Ortiz C, Lucena C, Lopez-Cabrera M, et al (1999) Extracellular HIV type 1 Tat protein induces CD69 expression through NF-kappaB activation: possible correlation with cell surface Tat-binding proteins. AIDS Res Hum Retroviruses 15: 1209–1218. 10480634 10.1089/088922299310304

[28] Shu L, Yin W, Zhuang H, Hua Z (2006) Comparison of gene expression profiles in mouse primary T cells under normal and prolonged activation. Blood Cells Mol Dis 37: 64–75. 16740399 10.1016/j.bcmd.2006.04.002

[29] Khorasanizadeh S (2004) The nucleosome: from genomic organization to genomic regulation. Cell 116: 259–272. 14744436 10.1016/s0092-8674(04)00044-3

[30] Luger K, Hansen JC (2005) Nucleosome and chromatin fiber dynamics. Curr Opin Struct Biol 15: 188–196. 15837178 10.1016/j.sbi.2005.03.006

[31] Jenuwein T, Allis CD (2001) Translating the histone code. Science 293: 1074–1080. 11498575 10.1126/science.1063127

[32] Rawlings JS, Gatzka M, Thomas PG, Ihle JN (2011) Chromatin condensation via the condensin II complex is required for peripheral T-cell quiescence. EMBO J 30: 263–276. 21169989 10.1038/emboj.2010.314 PMC3025460

[33] Dekker J, Rippe K, Dekker M, Kleckner N (2002) Capturing chromosome conformation. Science 295: 1306–1311. 11847345 10.1126/science.1067799

[34] Wang ZF, Krasikov T, Frey MR, Wang J, Matera AG, et al (1996) Characterization of the mouse histone gene cluster on chromosome 13: 45 histone genes in three patches spread over 1 Mb. Genome Res 6: 688–701. 8858344 10.1101/gr.6.8.688

[35] Nourshargh S, Marelli-Berg FM (2005) Transmigration through venular walls: a key regulator of leukocyte phenotype and function. Trends in immunology 26: 157–165. 15745858 10.1016/j.it.2005.01.006

[36] Dahl KN, Ribeiro AJ, Lammerding J (2008) Nuclear shape, mechanics, and mechanotransduction. Circulation research 102: 1307–1318. 18535268 10.1161/CIRCRESAHA.108.173989 PMC2717705

[37] Purushothaman D, Sarin A (2009) Cytokine-dependent regulation of NADPH oxidase activity and the consequences for activated T cell homeostasis. The Journal of experimental medicine 206: 1515–1523. 19546249 10.1084/jem.20082851 PMC2715083

[38] Westwood JA, Smyth MJ, Teng MW, Moeller M, Trapani JA, et al (2005) Adoptive transfer of T cells modified with a humanized chimeric receptor gene inhibits growth of Lewis-Y-expressing tumors in mice. Proceedings of the National Academy of Sciences of the United States of America 102: 19051–19056. 16365285 10.1073/pnas.0504312102 PMC1323148

[39] Gaspar-Maia A, Alajem A, Meshorer E, Ramalho-Santos M (2011) Open chromatin in pluripotency and reprogramming. Nature reviews Molecular cell biology 12: 36–47. 21179060 10.1038/nrm3036 PMC3891572

[40] Bolzer A, Kreth G, Solovei I, Koehler D, Saracoglu K, et al (2005) Three-dimensional maps of all chromosomes in human male fibroblast nuclei and prometaphase rosettes. PLoS biology 3: e157. 15839726 10.1371/journal.pbio.0030157 PMC1084335

