# Supplementary figures and images for: Developmental Heterogeneity in DNA Packaging Patterns Influences T-Cell Activation and Transmigration
Source: PLoS One. 2012 Sep 5;7(9):e43718. doi: 10.1371/journal.pone.0043718 (PMC3434176; doi:10.1371/journal.pone.0043718)

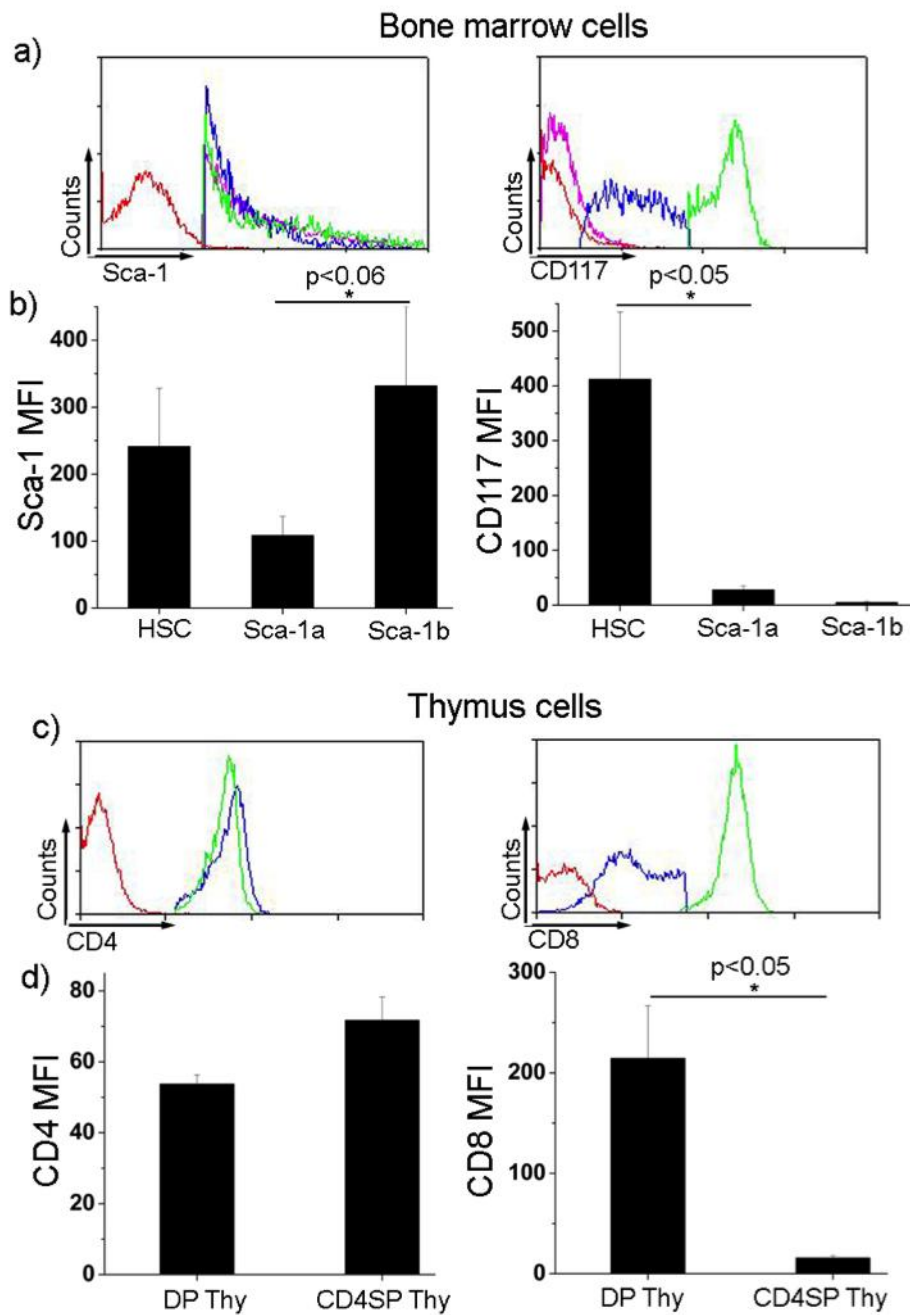

**Figure S1 Characterization of surface markers for bone marrow and thymic cells.**

Supplement: Figure S1 — Characterization of surface markers for bone marrow and thymic cells. a) Lineage deprived bone marrow cells were stained for HSC surface markers- Sca-1 and CD117. Representative flow plots showing the expression of these markers are shown. Sca-1a and Sca-1b populations were defined based on slightly different expression of Sca-1. Red- unstained, green- HSC, blue- Sca-1a, pink- Sca-1b. b) MFI averaged from three experiments for the surface markers as specified is plotted. The levels of Sca-1 and CD117 are significantly different in these populations. SE is plotted. c) Representative flow plots showing the expression of surface markers CD4 and CD8 in thymocytes. Red- unstained, green- DP thymocytes, blue- CD4 SP thymocytes. d) MFI averaged from three experiments for the surface markers as specified. Expression of CD4 is similar in DP and CD4 SP thymocytes, but the expression of CD8 is significantly reduced on CD4 SP thymocytes. SE is plotted. (PDF) [file pone.0043718.s001.pdf]

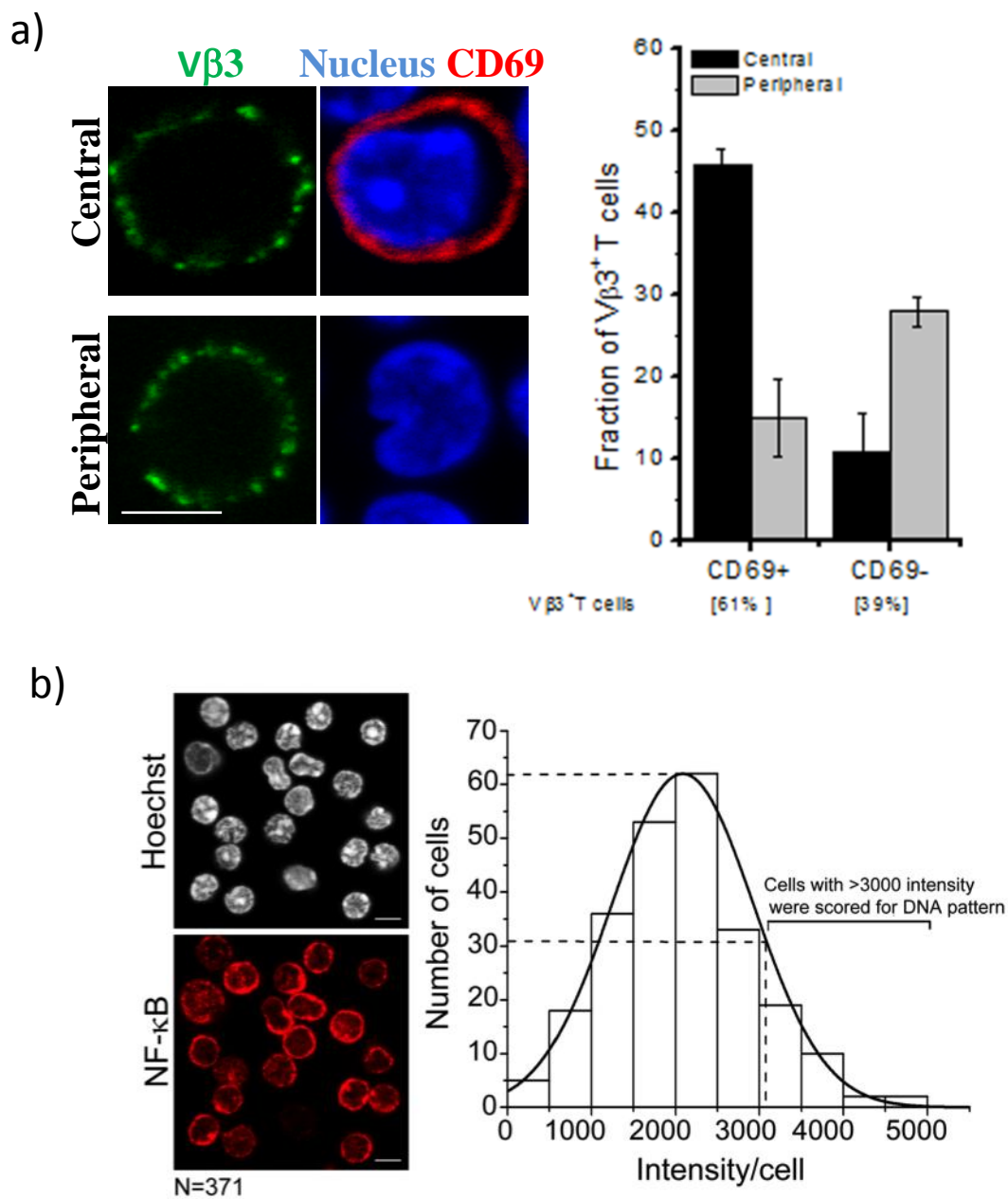

**Figure S3. Heterogeneity in DNA patterns influences early activation and gene expression.**

Supplement: Figure S3 — Heterogeneity in DNA patterns influences early activation and gene expression. a) Representative images of nuclei stained for DNA pattern with Hoechst 33342 (blue) in cells isolated from lymph node of mice challenged with SEA for 5 hrs, which are positive for Vβ3(green) and CD69(red). Scale bar 5 µm. Quantitative plot scoring for the two different DNA patterns in field images for Vβ3+ T cells and stained positive or negative for CD69 (n = 152). Error bars are standard deviation. b) Naïve T-cells were stained for NF-κB, counterstained with Hoechst and imaged. Representative confocal field views are shown. Scale bar 5 µm. The plot on right side highlights the cells that were above full width at half maxima. Only those cells were included in analysis of DNA pattern. (PDF) [file pone.0043718.s003.pdf]
