# Supplementary material for: Developmental Heterogeneity in DNA Packaging Patterns Influences T-Cell Activation and Transmigration
Source: PLoS One. 2012 Sep 5;7(9):e43718. doi: 10.1371/journal.pone.0043718 (PMC3434176; doi:10.1371/journal.pone.0043718)
Supplement: Figure S2 — Confirmation of heterochromatin patterns in T-cells by labelling various markers. (i). Representative field images of nuclei in thymocytes, CD4 naïve and memory cells, and activated T cells, showing the two different condensed DNA patterns observed during different stages of T cell differentiation by Hoechst staining. Central plane of the z-stack is shown. Central or peripheral pattern of DNA were counted from several such images. Scale bar 5 µm. (ii). a) Confirmation of DNA pattern in T cells was done by staining the cells with different nucleic binding dyes. Activated cells were treated with RNase A for 30 minutes, then stained with propidium iodide or sytox green. These dyes show co-localization with DNA stained with Hoechst. b) DNA that is stained brightly with Hoechst 33342 is also positive for HP1α, a heterochromatin binding protein. c) Naïve T cells were activated with αCD3-αCD134, a TNFR family co-stimulator molecule, or splenocytes from OT-II TCR transgenic mice were activated with maleylated ovalbumin for three days. T-cells were labelled by staining with αThy1.2, and the DNA imaged. The pattern of DNA organization is similar, irrespective of the kind of stimulation given to cells. Scale bar 2 µm. (iii). a) Plot indicating the two types of DNA patterns observed in CD8+ naïve T-cells (n = 230 cells each). (iv). a) Analysis of heterochromatin pattern in T-cells from blood: Representative images of nuclei stained for DNA pattern with Hoechst 33342 in cells isolated from blood which are positive for surface markers: CD4 (red) and CD62L (green). Scale bar 5 µm. b) Quantitative plot scoring for the two different DNA patterns in field images for cells isolated from blood which are stained positive for CD4 and CD62L. For comparison, naïve cell data from spleen cells is also shown. Error bars are standard error. (PDF) [file pone.0043718.s002.pdf]

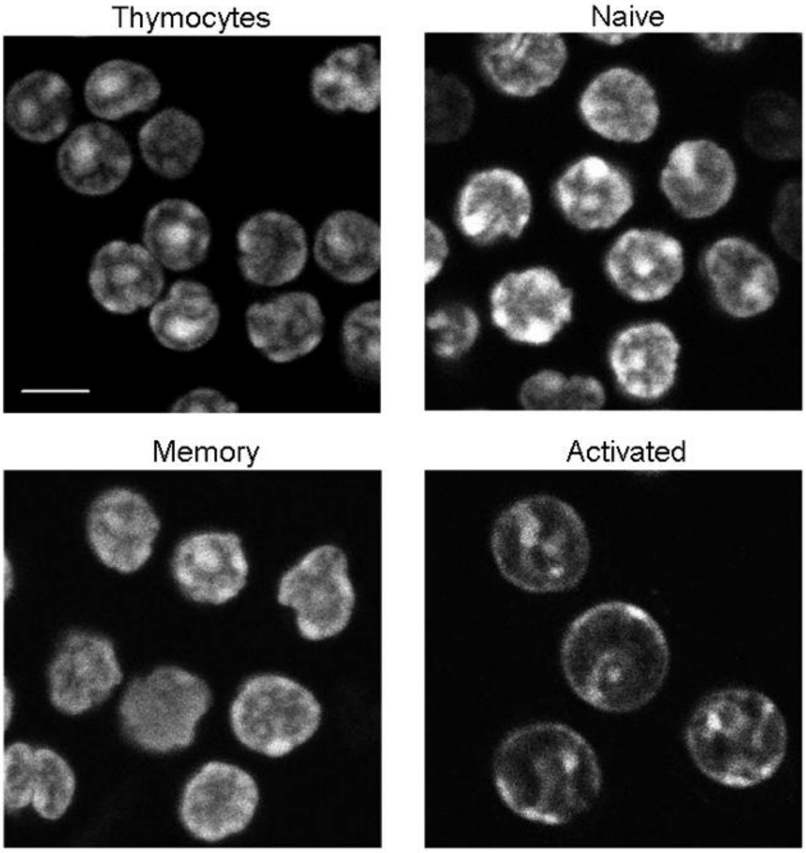

Figure S2(i). **Confirmation of heterochromatin patterns in T-cells by labelling various markers.**

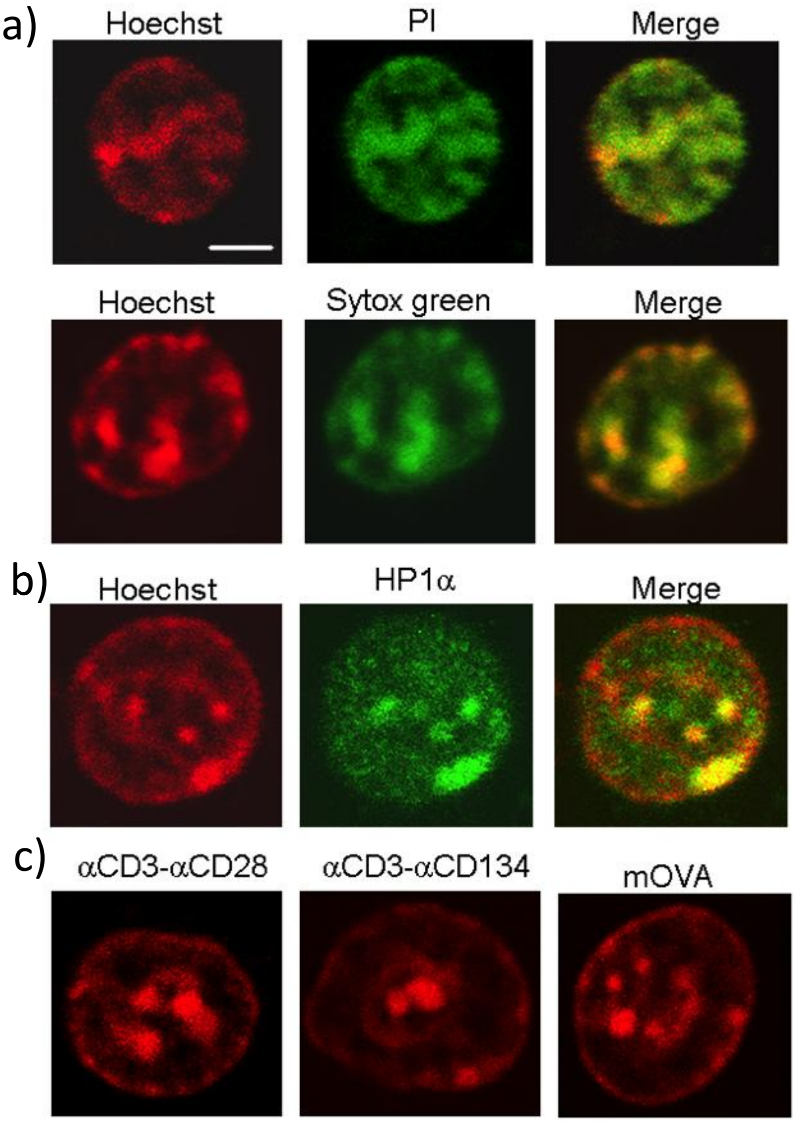

Figure S2(ii). **Confirmation of heterochromatin patterns in T-cells by labelling various markers.**

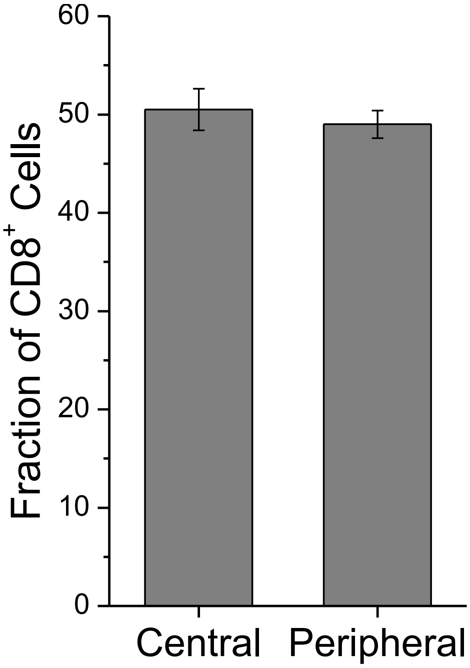

Figure S2(iii). **Confirmation of heterochromatin patterns in T-cells by labelling various markers.**

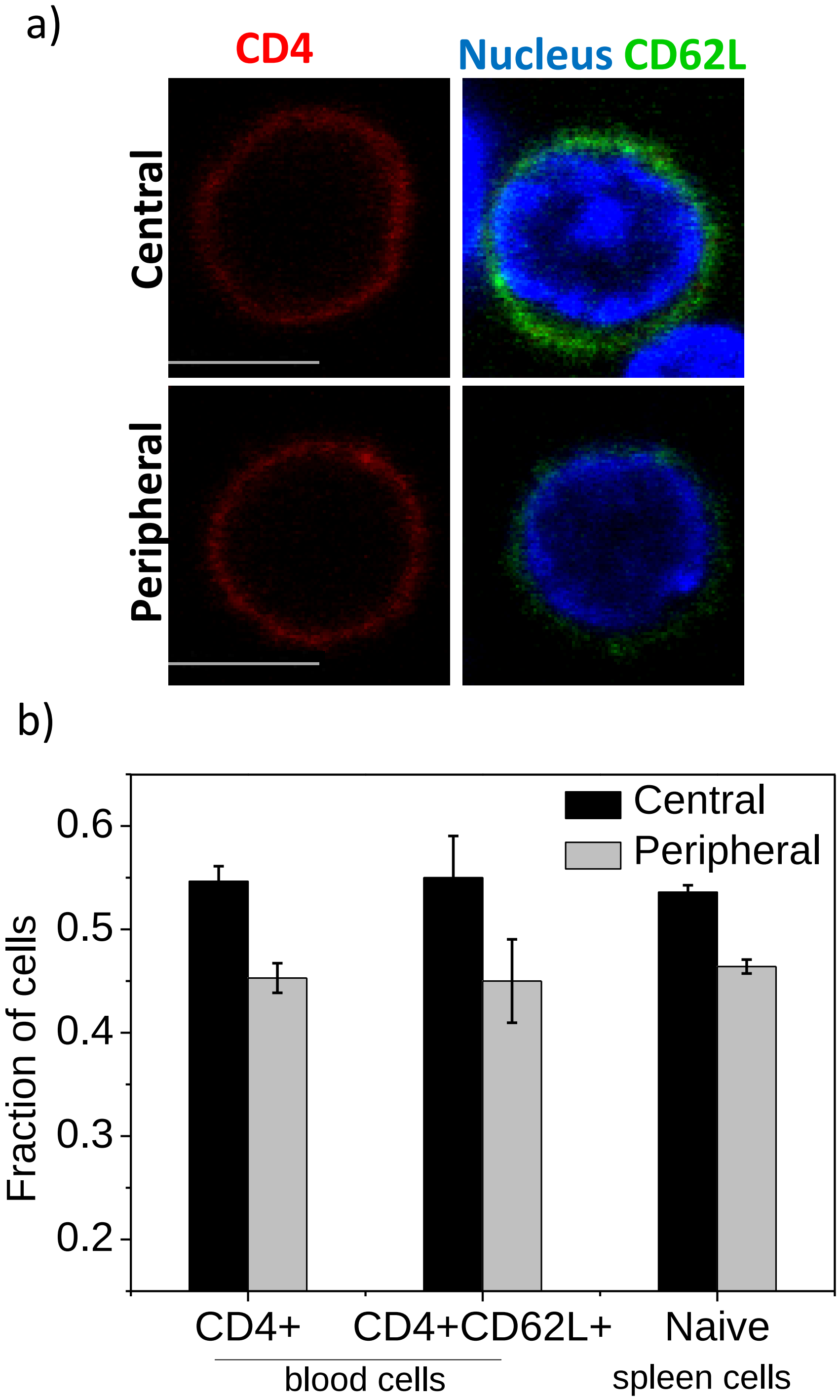

Figure S2(iv). **Confirmation of heterochromatin patterns in T-cells by labelling various markers.**
