# Supplementary material for: Developmental Heterogeneity in DNA Packaging Patterns Influences T-Cell Activation and Transmigration
Source: PLoS One. 2012 Sep 5;7(9):e43718. doi: 10.1371/journal.pone.0043718 (PMC3434176; doi:10.1371/journal.pone.0043718)
Supplement: Figure S4 — Characterization and analysis of heterochromatin patterns in activated T-cells. (i). Naïve T-cells were activated for 48 hours with αCD3-αCD28 antibody coated eads in absence (D2) or presence of TSA (TSA treated D2 cells). Cells were then stained with αCD69 or αCD25 to confirm activation with the antibody coated beads. Histone deacetylase inhibitor TSA was used as an additional control to assess the role of chromatin perturbation and T-cell activation. (ii). Cell cycle analysis for day 2 activated cells. G0–G1, S and G2 phase have 56.9%, 18.49% and 14.7% cells, respectively. Less than 50% of cells have entered cell cycle. (iii). a) Representative images and quantitative plot scoring for the two different DNA patterns in field images for single positive (SP) resting, activated and D7 thymocytes (n = 100 cells). b) Representative images of naïve and SP thymocytes maintained in IL-7 for 4 days and the plot indicating the differences in DNA patterns (n = 50 cells each). (PDF) [file pone.0043718.s004.pdf]

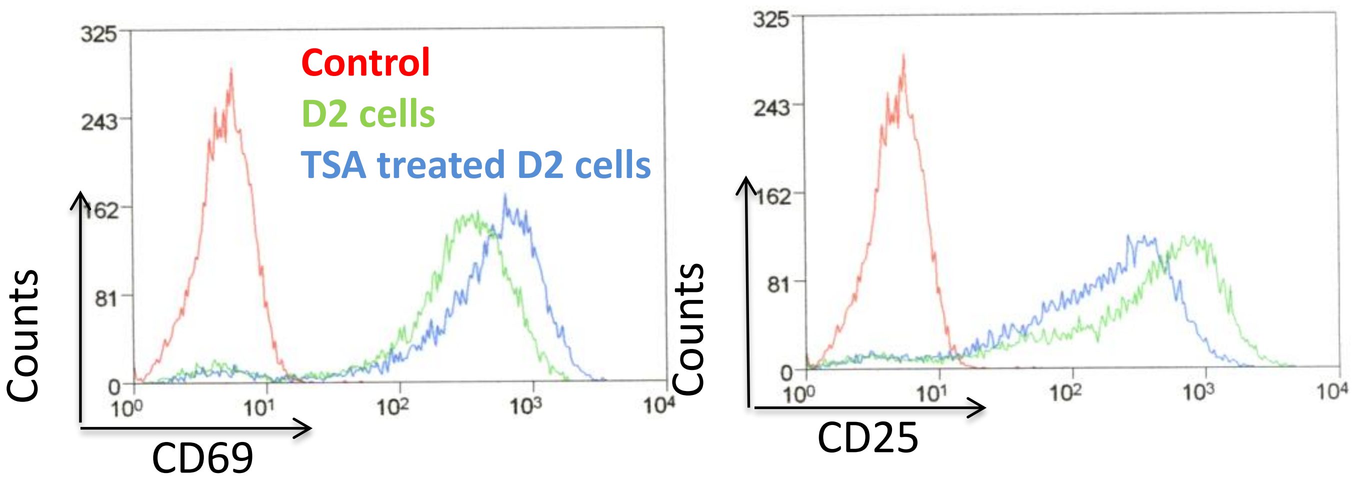

Figure S4(i). **Characterization of and analysis of heterochromatin patterns in activated T-cells.**

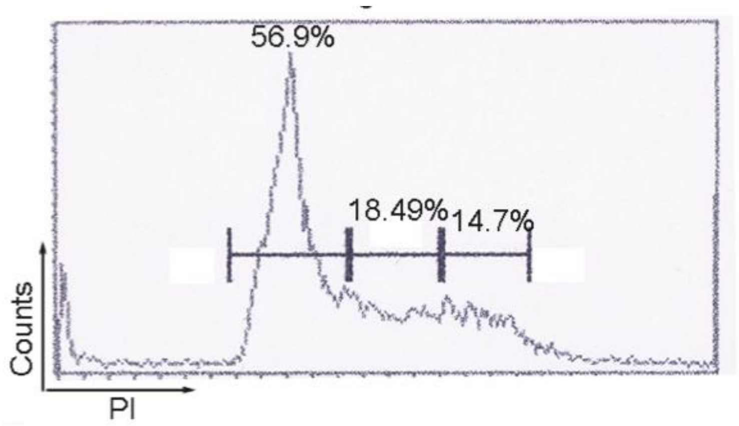

Figure S4(ii). **Characterization of and analysis of heterochromatin patterns in activated T-cells.**

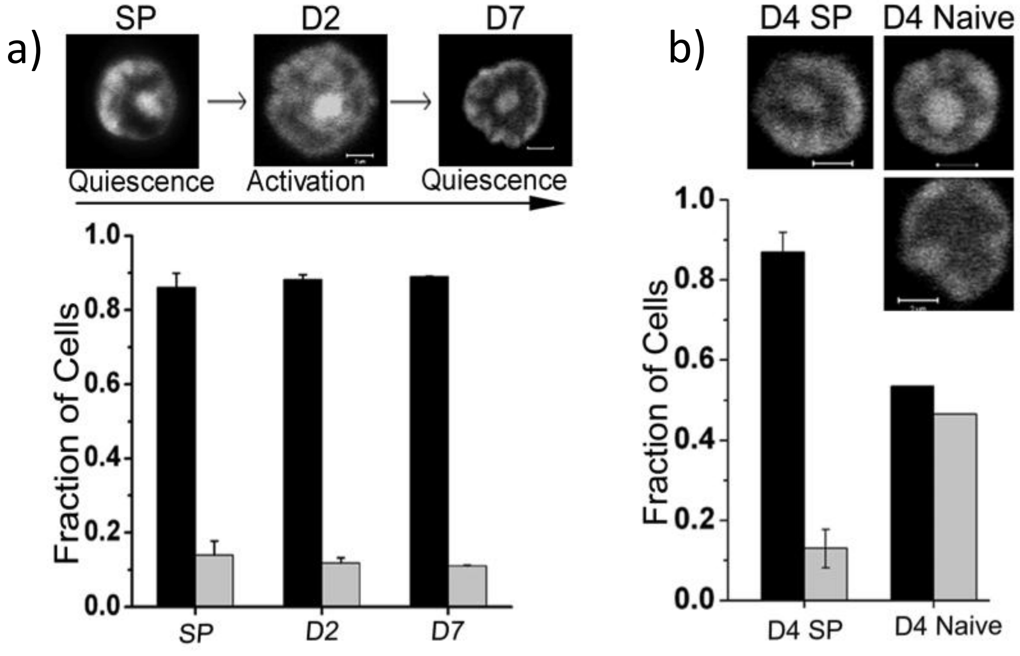

Figure S4(iii). **Characterization of and analysis of heterochromatin patterns in activated T-cells.**
