# Supplementary material for: Developmental Heterogeneity in DNA Packaging Patterns Influences T-Cell Activation and Transmigration
Source: PLoS One. 2012 Sep 5;7(9):e43718. doi: 10.1371/journal.pone.0043718 (PMC3434176; doi:10.1371/journal.pone.0043718)
Supplement: Figure S5 — Histone modifications changes during T-cell development. (i). MFI data and representative flow plots showing the histogram of H3Ac, H3K4me2, H3K9Ac, H3K9me, H3K27me3 and background staining (inset) in T–cell subsets from bone marrow (BM), thymus, and spleen. The MFI plots for the various histone antibodies were calculated by subtracting the background control values obtained in the respective population. The levels of these modifications (including background) change during differentiation, being higher in bone marrow cells but lower in thymic cells and increasing again during T-cell activation. (ii). Collage of H3Ac staining in a) naïve T-cells b) activated cells and c) rabbit secondary antibody control images. Scale bar 5 µm. Collage of images showing HP1α staining in d) naïve cells and e) activated cells. Scale bar 2 µm. (iii). a) H3Ac staining (green) in naïve and D2 cells, DNA counterstained with Hoechst (red) showing the spatial reorganization of H3Ac. b) Representative DNA images of CD4+ naïve T-cells activated for 48 hours with αCD3-αCD28 antibody in presence of 10 nM TSA. Scale bar 2 µm. c) Quantitative plot scoring for the two different DNA patterns in field images of naïve and TSA treated (5 nM, 10 nM) or control activated T cells (T48TSA0) (n = 1000 cells each). (PDF) [file pone.0043718.s005.pdf]

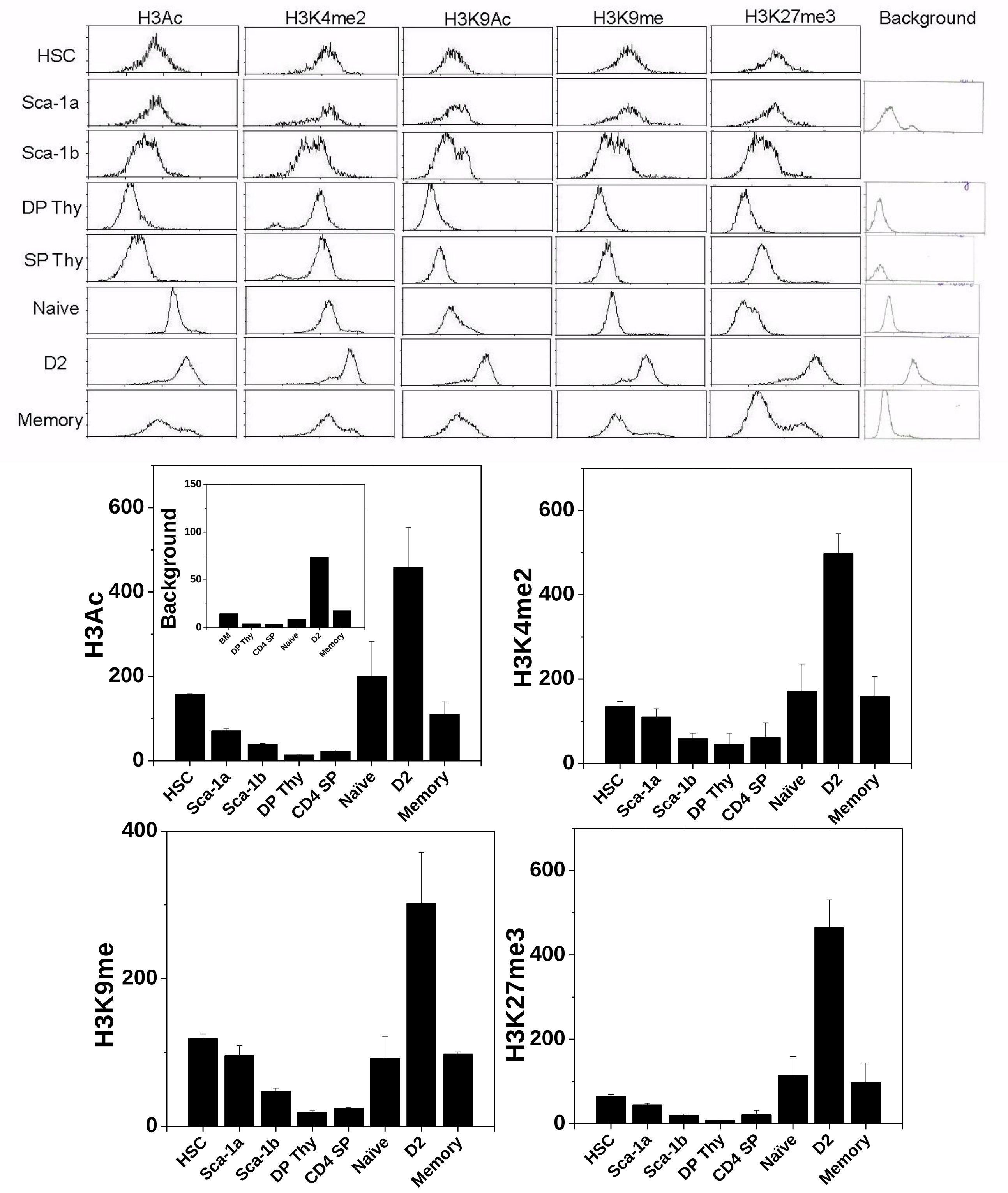

Figure S5(i). **Histone modifications changes during T-cell development.**

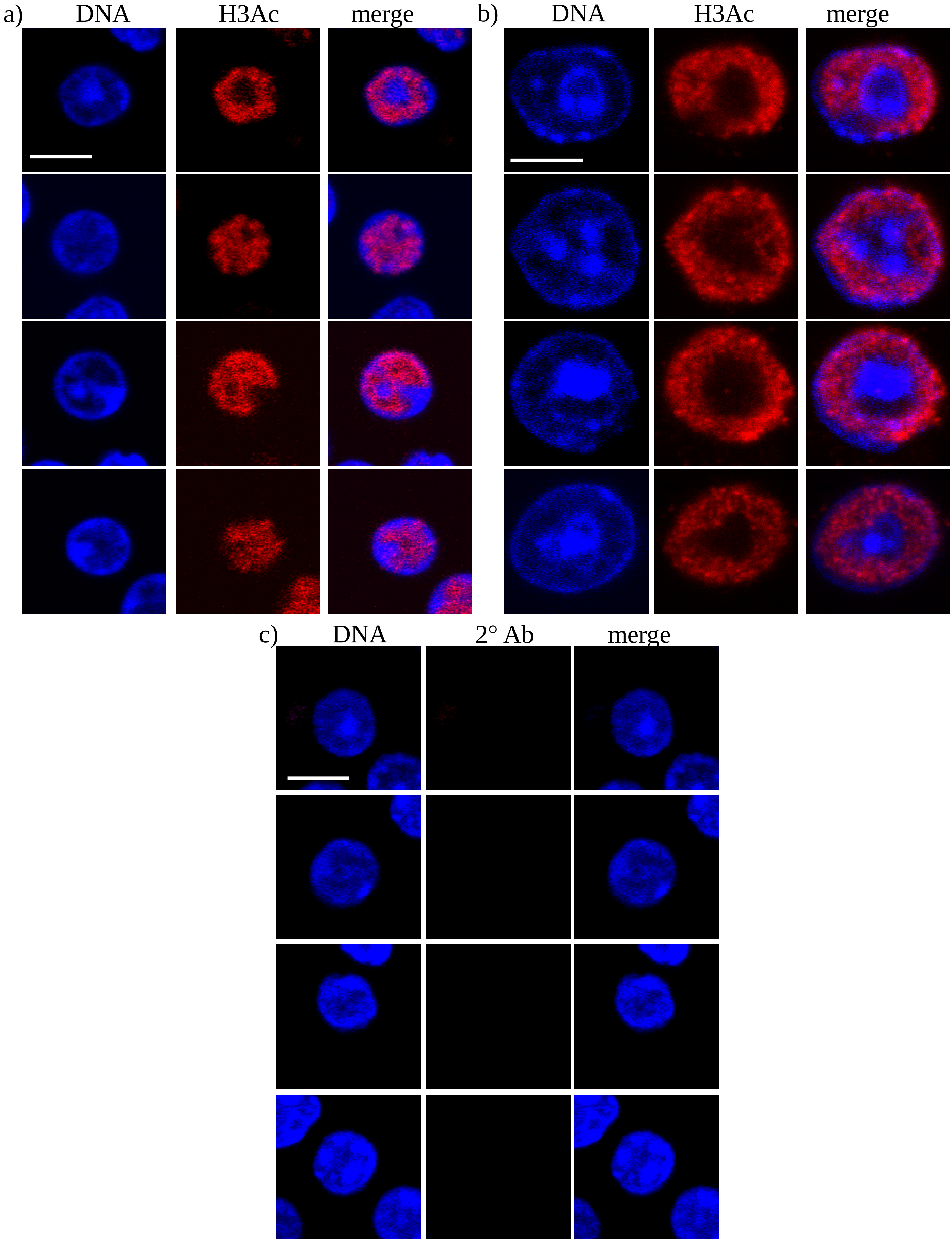

Figure S5(ii). **Histone modifications changes during T-cell development.**

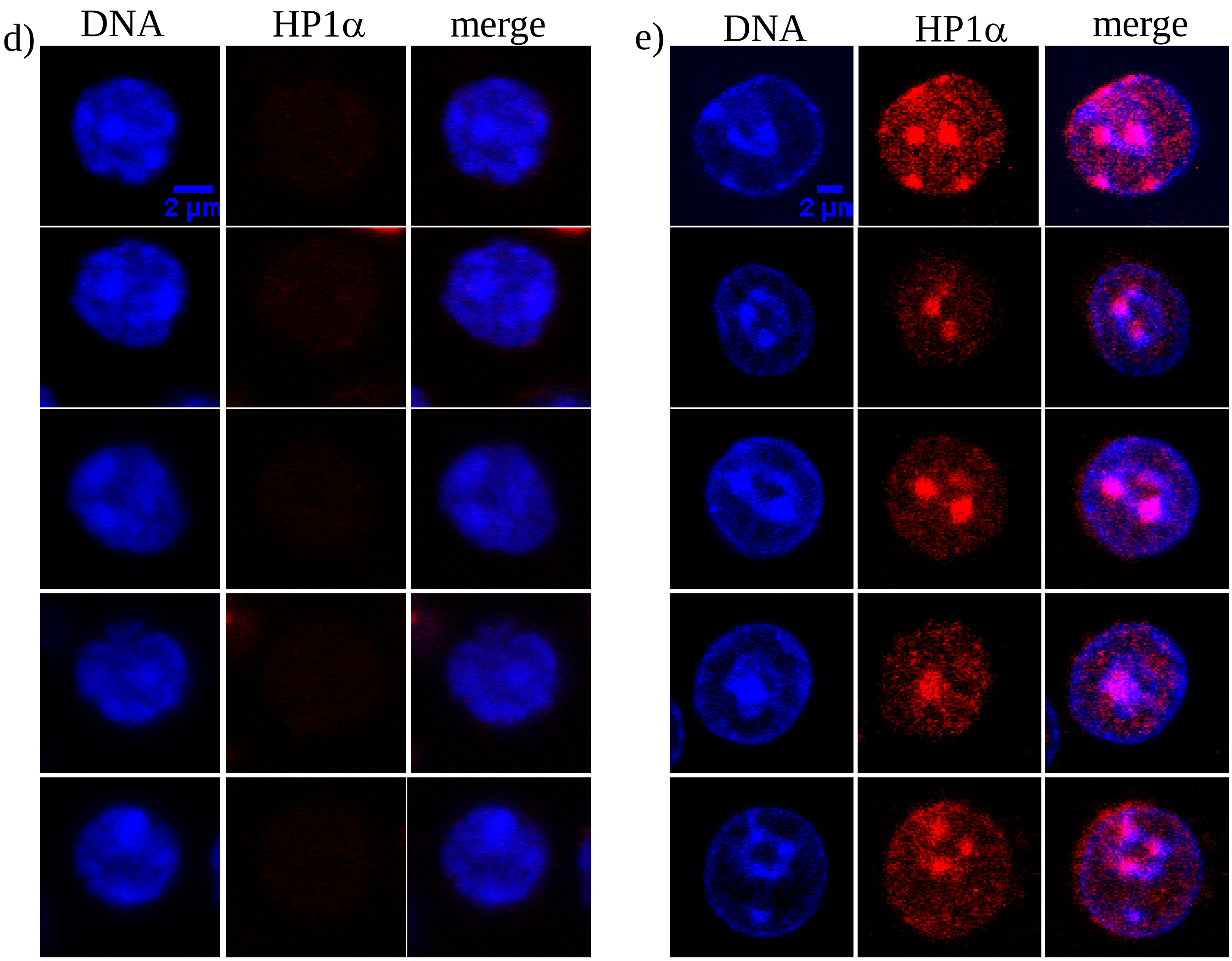

Figure S5(ii). **Histone modifications changes during T-cell development.**

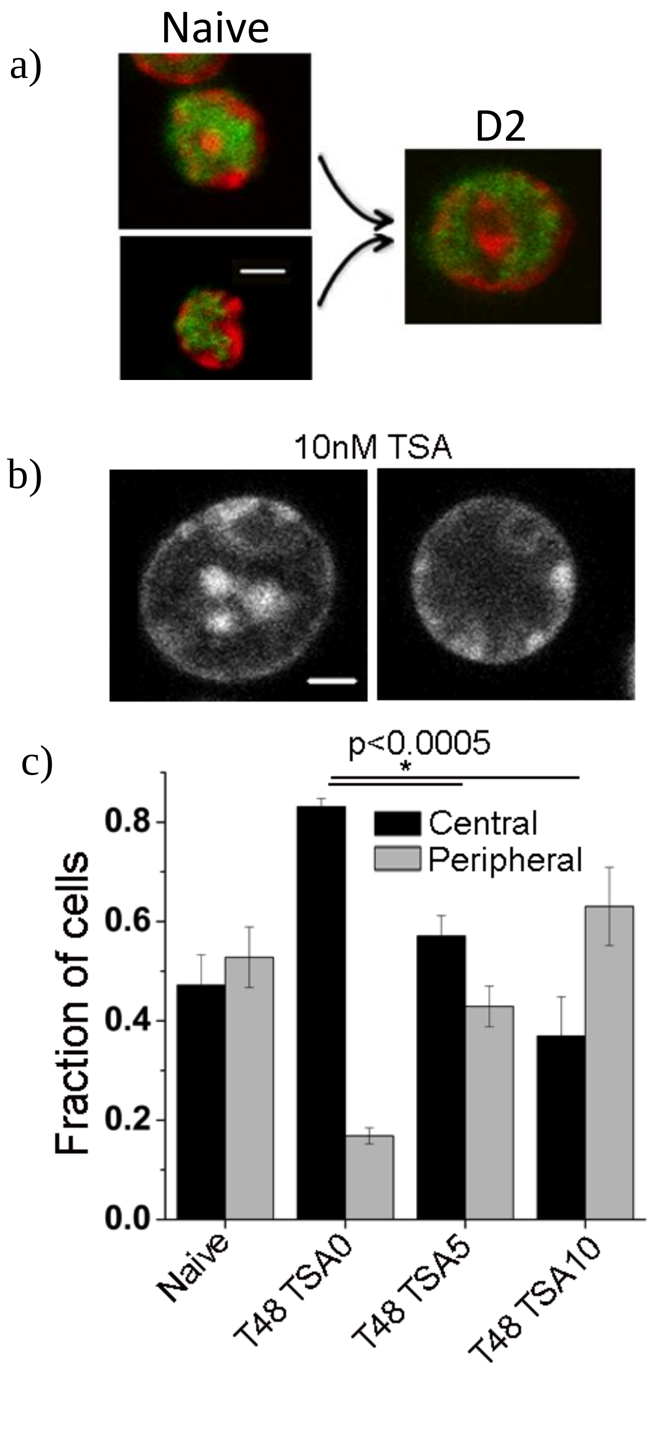

Figure S5(iii). **Histone modifications changes during T-cell development.**
