# Supplementary material for: Developmental Heterogeneity in DNA Packaging Patterns Influences T-Cell Activation and Transmigration
Source: PLoS One. 2012 Sep 5;7(9):e43718. doi: 10.1371/journal.pone.0043718 (PMC3434176; doi:10.1371/journal.pone.0043718)
Supplement: Figure S6 — Chromosomal positions and gene expression analysis in naïve and activated T-cells. (i). a) Normalized average distance of centroid of chromosomes from the nuclear centroid (n = 70). Inset- z-projection of DNA (blue) and chromosome paint for chromosome pair 1 (green) and 4 (red). Upon activation, chromosome 1 repositioned towards the nuclear periphery (0.86) as compared to the naïve cells where it was present at 0.76 relative to the nuclear centroid. Chromosomes 3, 4 and 17 also repositioned to more peripheral positions during T-cell activation. Scale bar 2 µm. b) Colour graph representing expression of genes on four candidate chromosomes in naïve and activated cells as observed by microarray data. Darker shades represent lower intensity; lighter shades represent higher intensity. (ii). a) Cytoskeleton is an important component in maintaining cell shape and size, and T-cell activation is accompanied by an increase in cell and nuclear size. Differential expression of cytoskeleton related genes in naïve and activated (D2) cells as analyzed by microarray. Higher expression of most cytoskeletal genes by activated cells probably explains the higher nuclear volume of those cells. Gene names are given on the left side. Cooler colors represent lower expression value; warmer colors represent higher expression value. b) Microtubules were stained by α-tubulin antibody and z-stacks were acquired. Shown in the image are z-projections of microtubule staining in naïve and activated T cells. Scale bar 2 µm. Staining for lamin B1, major component of nucleoskeleton, appears similar in naïve and activated cells, however, it appears indeted in naïve cells but smooth and continuous in activated cells. Scale bar 5 µm. (PDF) [file pone.0043718.s006.pdf]

a)

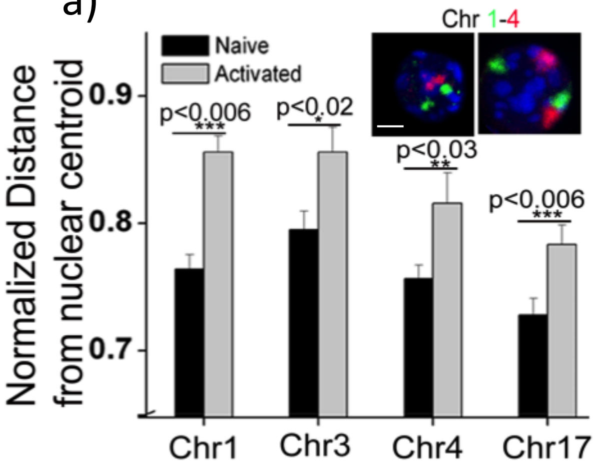

b)

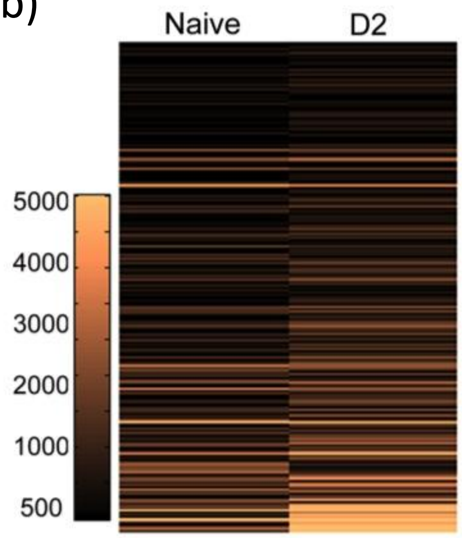

Figure S6(i). **Chromosomal positions and gene expression analysis in naïve and activated T-cells.**

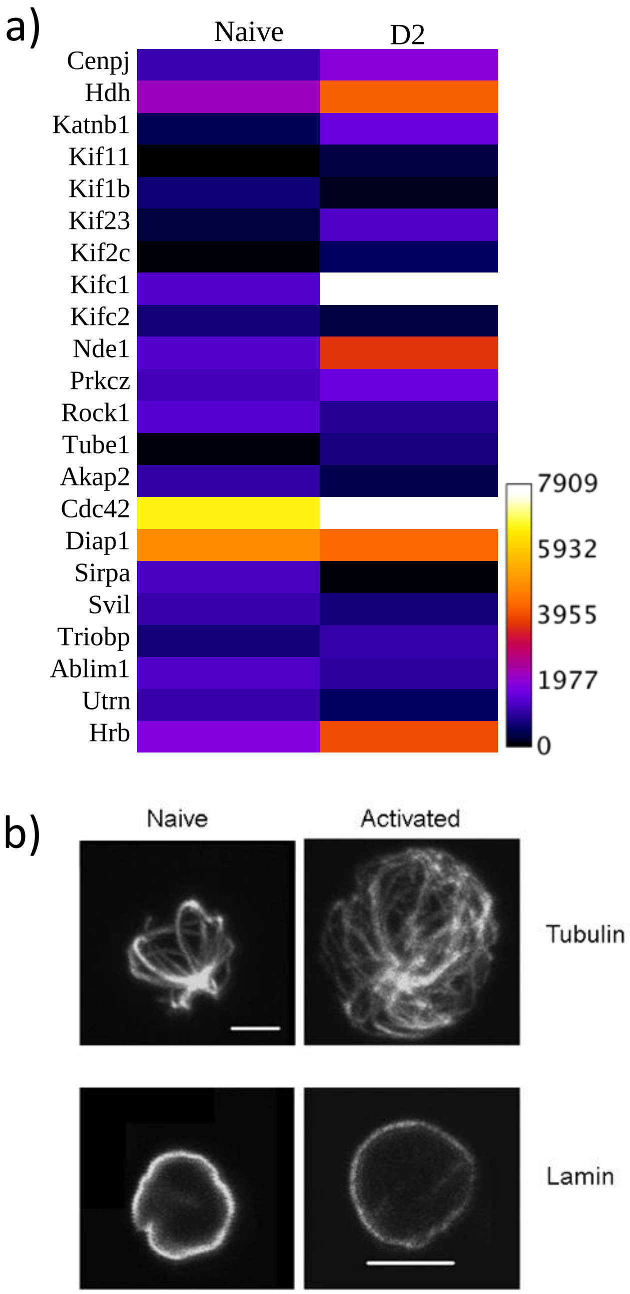

Figure S6(ii). **Chromosomal positions and gene expression analysis in naïve and activated T-cells.**
