# Supplementary material for: Developmental Heterogeneity in DNA Packaging Patterns Influences T-Cell Activation and Transmigration
Source: PLoS One. 2012 Sep 5;7(9):e43718. doi: 10.1371/journal.pone.0043718 (PMC3434176; doi:10.1371/journal.pone.0043718)
Supplement: Figure S7 — 3C analysis of histone gene cluster during T-cell development. (i). A) Map of histone cluster I on chromosome 13. The rectangles show the position and size of each patch of histone genes in the cluster. The down arrows indicate the position of Xho I sites selected. The short horizontal arrows show the position of the primers used. The thick red arrows show the positions of the H3 genes within the cluster. The circles show the sites considered in the schematic of the association of the H3 genes. B) PCR for 3C for the histone gene cluster in mouse cells with different primer combinations, T: thymocyte, N: Naïve and D: D2 activated T cells: Lanes 1,18: 1 kb DNA Ladder; 2: control 1 (without crosslinking, digestion and ligation); lane 3: control 2 (without crosslinking and ligation); lane 4: control 3 (without crosslinking); lane 5: control 4 (without ligation); Lane 6, 7, 8: Primers 1 and 5; lane 9, 10, 11: Primers 2 and 5; lane 12, 13, 14: Primers 2 and 17; lane 15,16, 17: Primers 1 and 3. C) Lane 1, 15: 1 kb ladder; lane 2–5: controls; lane 6,7,8: primers 5 and 6; lane 9,10,11: primers: 5 and 9; lane 12,13,14: primers 5 and 17. D) Cartoon depicting the associations of encircled regions in schematic in panel A in thymocytes, CD4+ naïve and activated T cell. (ii). 3C control experiments: with different primer combinations. A: 1–16: −crosslink, −digesion, −ligation, lane 17: 1 kb ladder. B: 1–7: crosslink, −digesion, −ligation; 8, 18: marker, 9–17: −crosslink, +Xho I, −ligase. C: 1–12: −crosslink, +Xho I, −ligase; 13–16: −crosslink, +Xho I+ligase, 17: marker. D: 1–17: −crosslink, +Xho I+ligase, 18 marker. E: 1–17: +crosslink, +digestion, −ligation, 18; marker. F: 1–8: +crosslink, +digestion, −ligation, 9; marker. (iii). 3C analysis of thymocyte cells shown in a–d. a) lanes 1–10: primers 1F and all the reverse primers; 12–18: primers 5F and all the reverse primers. b) 1–9: primers 2F and all reverse primers;11–18: primers 3F and all reverse primers. c)1–6: primers 6F and [file pone.0043718.s007.pdf]

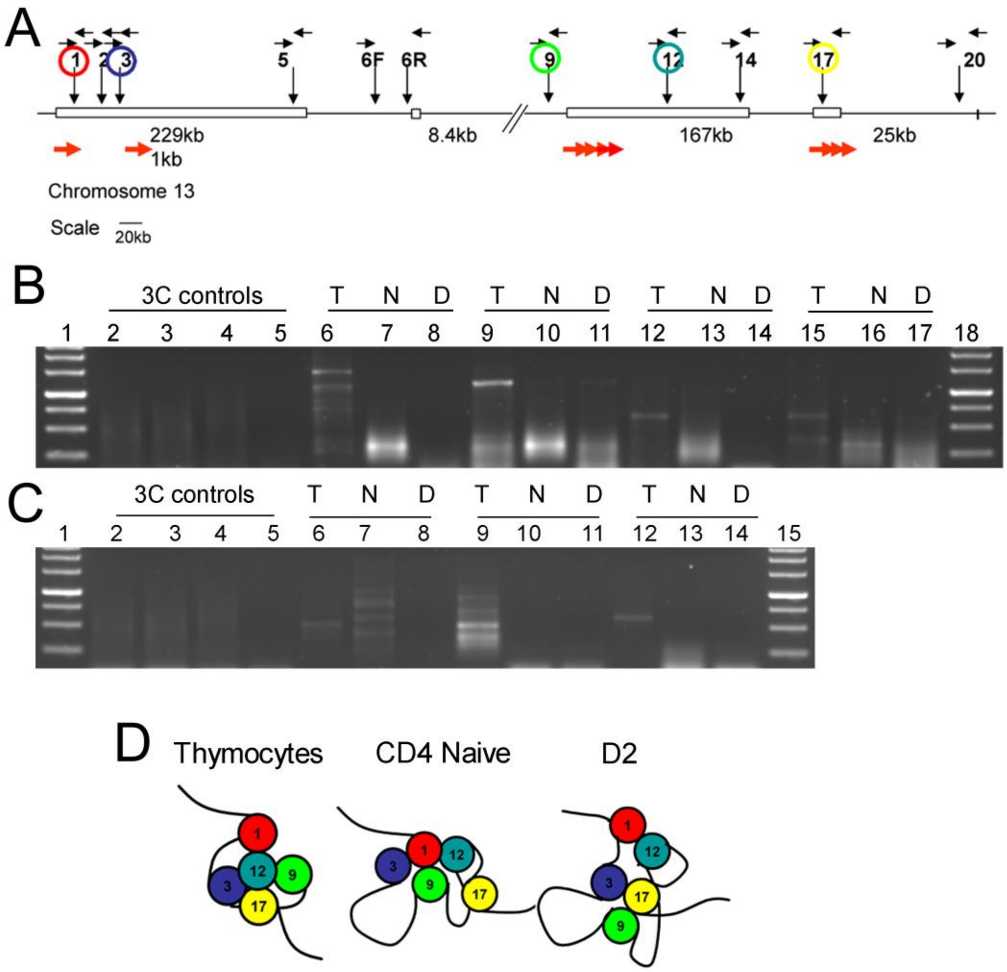

Figure S7(i). **3C analysis of histone gene cluster during T-cell development.**

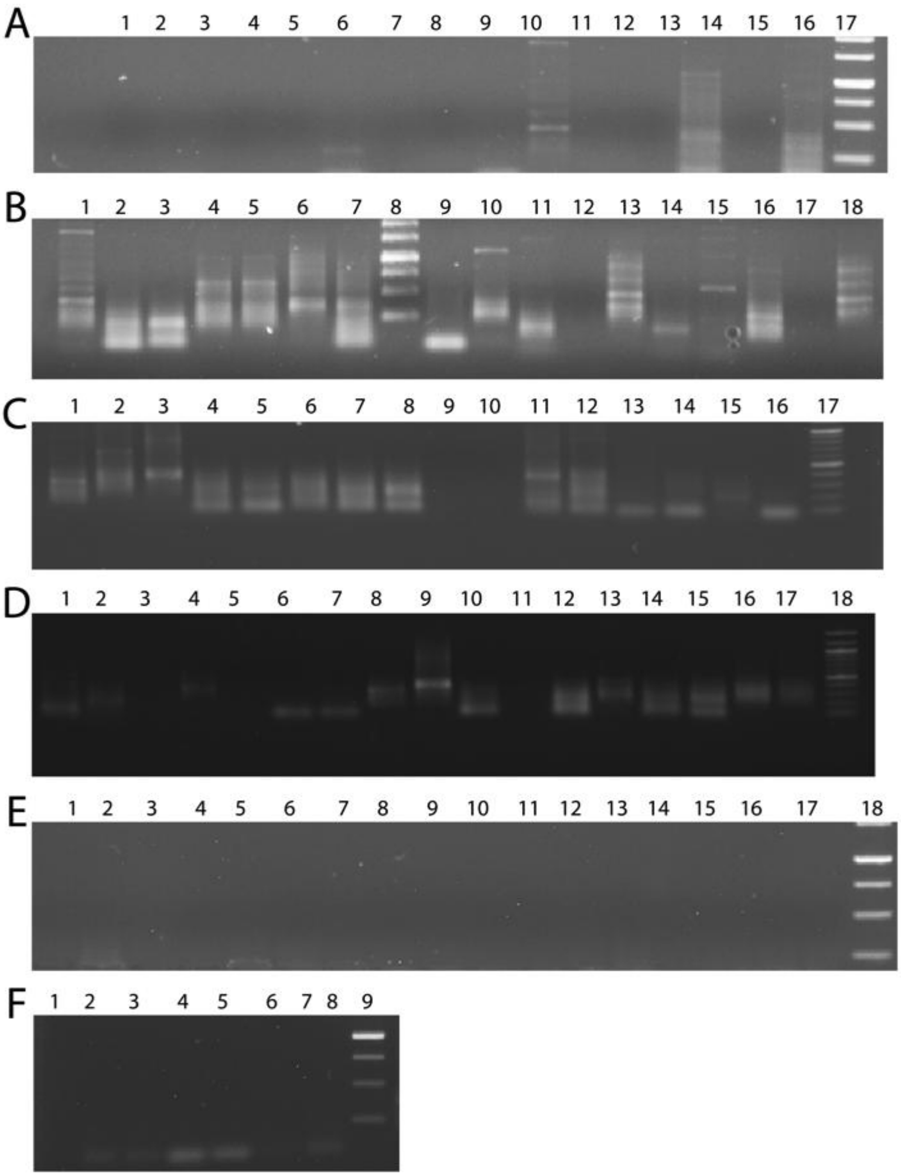

Figure S7(ii). **3C analysis of histone gene cluster during T-cell development.**

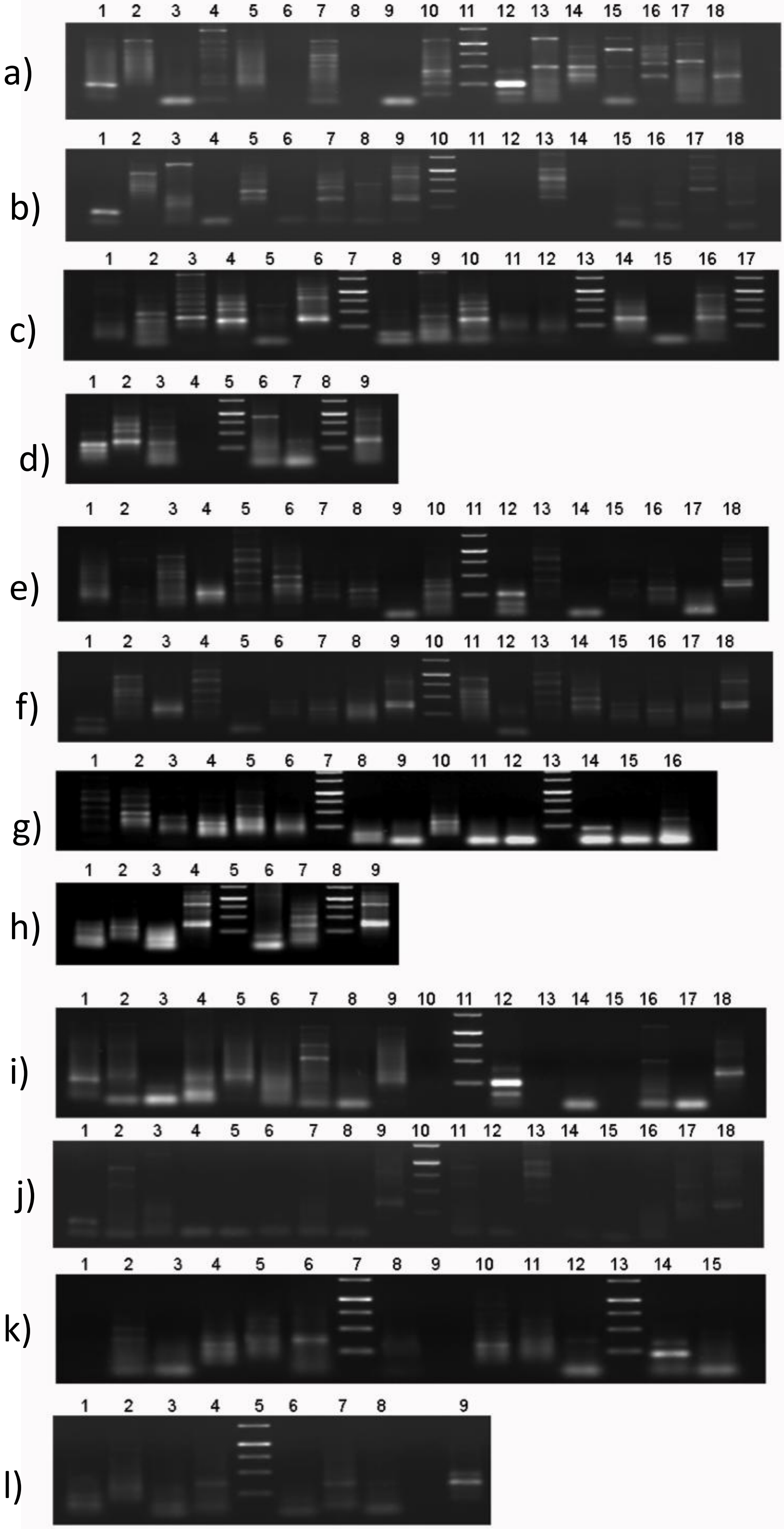

Figure S7(iii). **3C analysis of histone gene cluster during T-cell development.**

a)

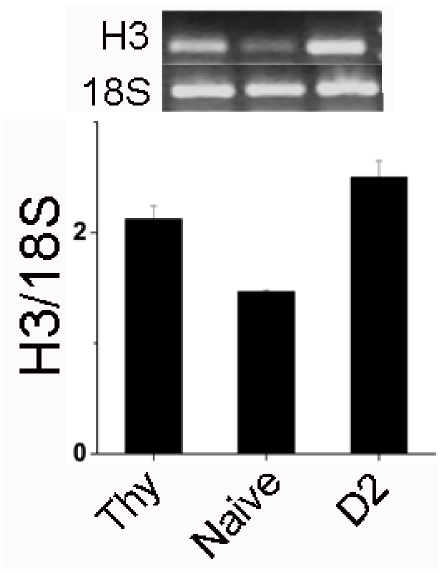

b)

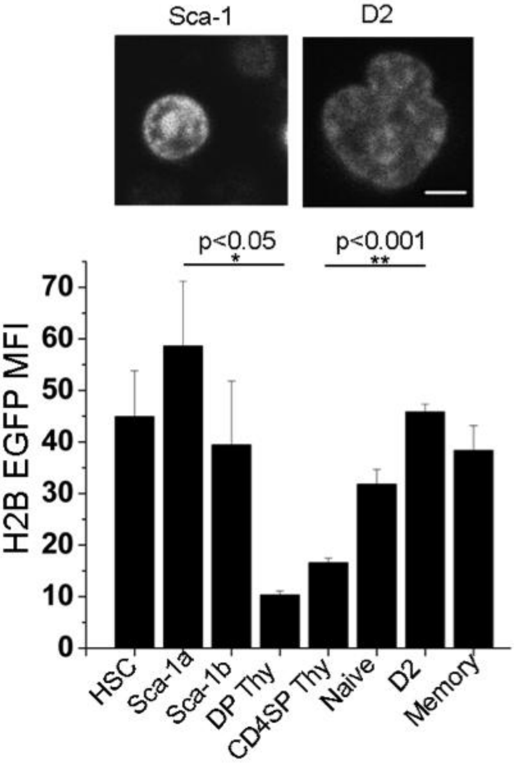

Figure S7(iv). **3C analysis of histone gene cluster during T-cell development.**
