# Supplementary material for: Developmental Heterogeneity in DNA Packaging Patterns Influences T-Cell Activation and Transmigration
Source: PLoS One. 2012 Sep 5;7(9):e43718. doi: 10.1371/journal.pone.0043718 (PMC3434176; doi:10.1371/journal.pone.0043718)
Supplement: Figure S8 — Implications of heterogeneity in DNA patterns on naive T-cells functions. (i). a) Representative field-view images of naïve T-cells without (Control) and with different compressive loads (Load1 and Load 2). b) Quantitative plot scoring for the two different DNA patterns in field images in naïve T-cells without (Control) and with higher compressive load of 2 nN (Load2) for increase in aspect ratio (n = 500 cells each). c) Quantitative graph showing the recovery of GFP+ cells in lymph node and spleen at later time points (72 hr and 7days post adoptive transfer) and analyzed for DNA patterns. (ii). Histogram plot showing the nuclear aspect ratio for cells with central or peripheral pattern of condensed DNA, without (Control) and with compressive load (Load). (iii). Fraction of GFP+ naïve T-cells in blood with central and peripheral DNA patterns after 15 hr and 7 days of adoptive transfer into wild type congenic mice. Mean+ SD is plotted. (PDF) [file pone.0043718.s008.pdf]

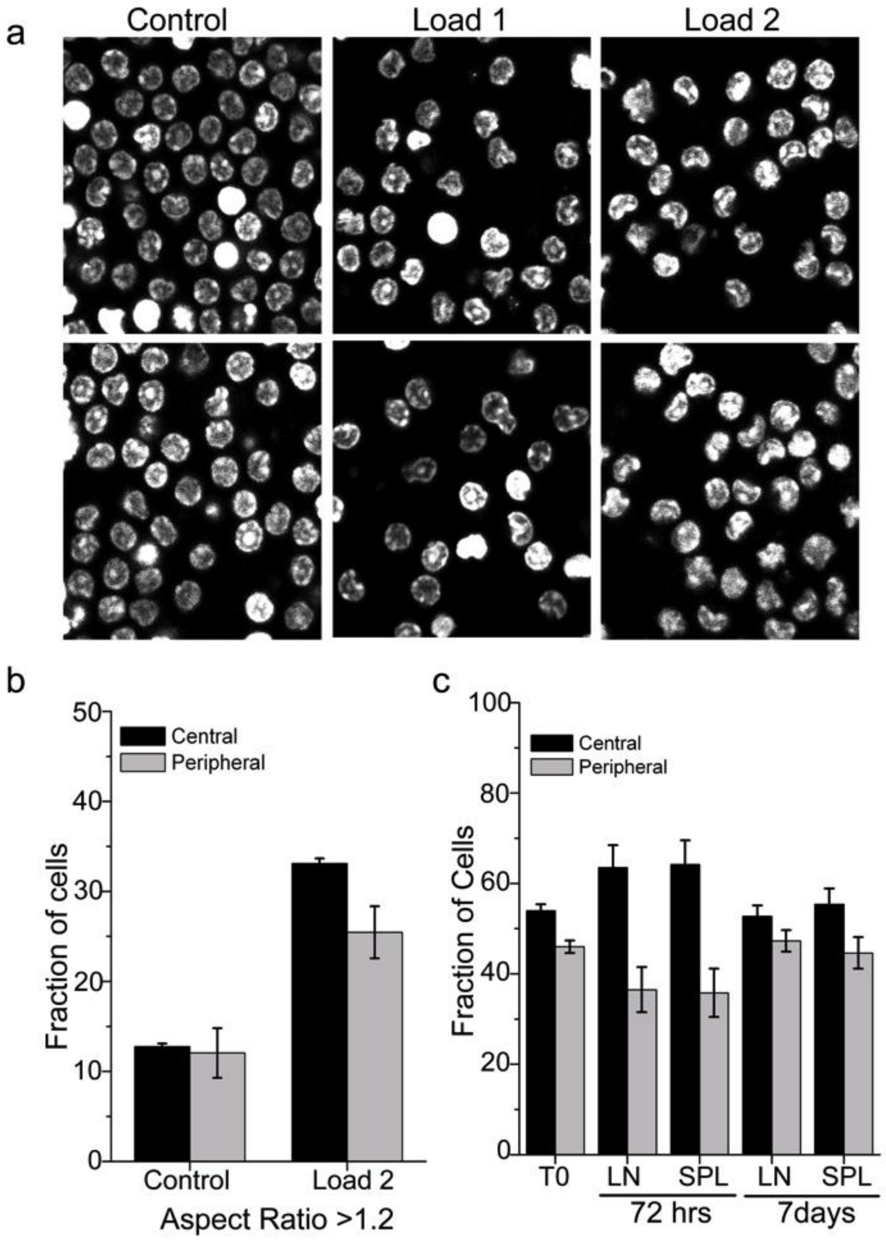

Figure S8(i) **Implications of heterogeneity in DNA patterns on naive T-cells functions.**

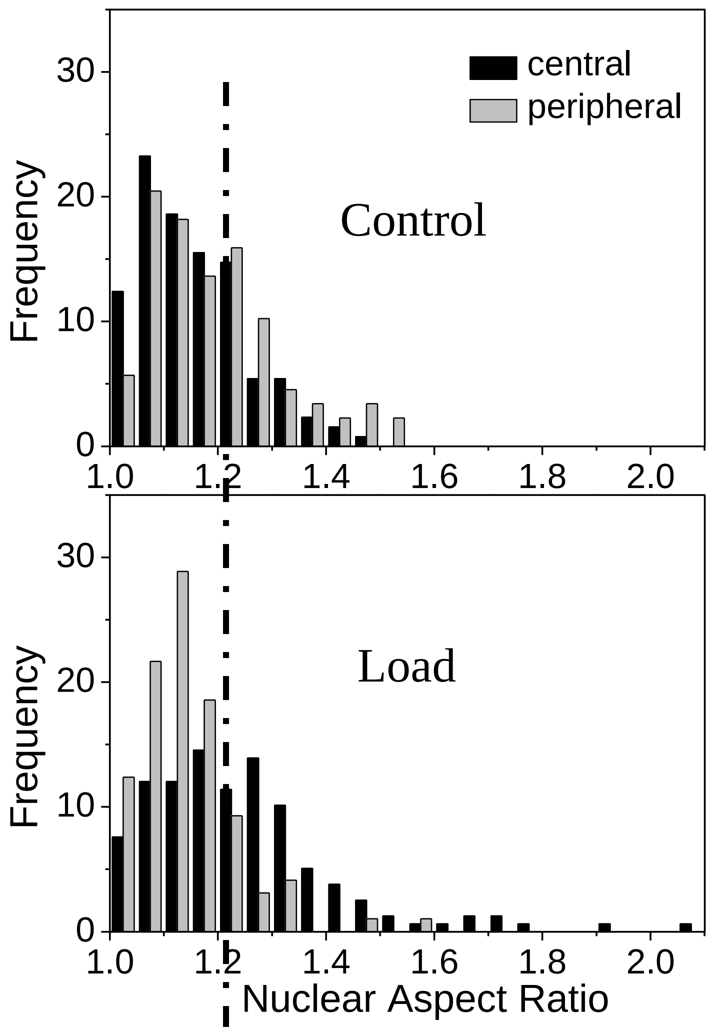

Figure S8(ii) **Implications of heterogeneity in DNA patterns on naive T-cells functions.**

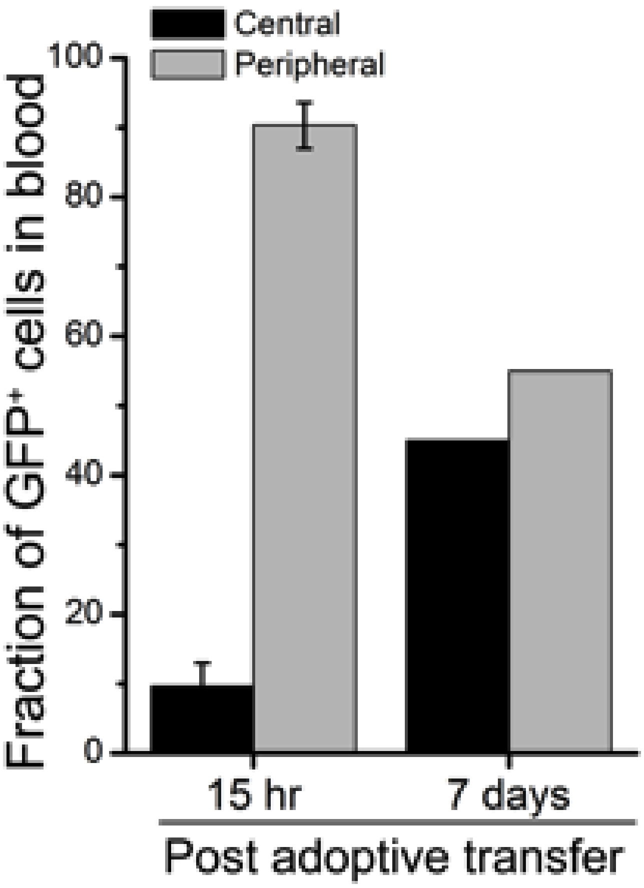

Figure S8(iii) **Implications of heterogeneity in DNA patterns on naive T-cells functions.**
