# Supplementary material for: Developmental Heterogeneity in DNA Packaging Patterns Influences T-Cell Activation and Transmigration
Source: PLoS One. 2012 Sep 5;7(9):e43718. doi: 10.1371/journal.pone.0043718 (PMC3434176; doi:10.1371/journal.pone.0043718)
Supplement: Table S1 — Table with list of genes located on chromosomes 1,3,4, and 17 and which show differential activity in naïve and activated T-cells. (PDF) [file pone.0043718.s012.pdf]

**Supporting Table 1.** Table with list of genes located on chromosomes 1,3,4, and 17 and which show differential activity in naïve and activated T-cells.

| Gene name | Log2 fold change (activated/naïve) | Chromosomal location |
|---|---|---|
| Psrc1 | 5.290852095 | chr3 |
| E130016E03Rik | 5.013259986 | chr4 |
| AK017340 | 4.877883679 | chr4 |
| Exo1 | 4.520158443 | chr1 |
| Ect2 | 4.489326193 | chr3 |
| Kif2c | 4.485699979 | chr4 |
| Melk | 4.470413467 | chr4 |
| Rad54l | 4.346270238 | chr4 |
| Sgol2 | 4.179244119 | chr1 |
| NAP101638-1 | 4.15270038 | chr1 |
| Dtl | 3.924946994 | chr1 |
| 2700049P18Rik | 3.887160796 | chr1 |
| Nek2 | 3.730091558 | chr1 |
| Nsl1 | 3.72053413 | chr1 |
| 1810007P19Rik | 3.626706075 | chr4 |
| Orc1l | 3.420240344 | chr4 |
| Chtf18 | 3.298900909 | chr17 |
| Ctla4 | 3.277094113 | chr1 |
| Ndc80 | 3.234565424 | chr17 |
| Zranb3 | 3.230940627 | chr1 |
| C230078M08Rik | 3.140097928 | chr17 |
| Fdps | 3.042654303 | chr3 |
| Paqr4 | 3.022469592 | chr17 |
| F730047E07Rik | 2.862855332 | chr4 |
| Prim2 | 2.783644392 | chr1 |
| Cchcr1 | 2.779356879 | chr17 |
| Kifc1 | 2.566579456 | chr17 |
| E130306D19Rik | 2.478283868 | chr4 |
| Als2cr4 | 2.450124015 | chr1 |
| Smc2 | 2.325604671 | chr4 |
| Tmem48 | 2.239530909 | chr4 |
| Lxn | 2.118882963 | chr3 |
| Gpsm2 | 2.097323972 | chr3 |
| Rdbp | 2.068272875 | chr17 |
| 4930579G24Rik | 2.05919406 | chr3 |
| 1700025G04Rik | 2.05837122 | chr1 |
| Wdr90 | 1.977370371 | chr17 |
| Nme7 | 1.94222501 | chr1 |
| Rpusd1 | 1.893521499 | chr17 |
| 6720467C03Rik | 1.888061341 | chr4 |
| Psmb2 | 1.843279374 | chr4 |

| | | |
|---|---|---|
| 9130206I24Rik | 1.837543503 | chr4 |
| Dclk2 | 1.759182422 | chr3 |
| Fancg | 1.747447774 | chr4 |
| Nans | 1.737913578 | chr4 |
| Wnt10a | 1.73243374 | chr1 |
| Rcc1 | 1.692891046 | chr4 |
| 2310030N02Rik | 1.668418375 | chr4 |
| Mfsd2 | 1.642707305 | chr4 |
| Msh6 | 1.634959463 | chr17 |
| Nmnat1 | 1.623911949 | chr4 |
| Lgtn | 1.621644063 | chr1 |
| 1810054D07Rik | 1.619295781 | chr4 |
| Nbn | 1.581630469 | chr4 |
| 6230416J20Rik | 1.564329963 | chr4 |
| Hnrpll | 1.557985897 | chr17 |
| Nrm | 1.529396256 | chr17 |
| Cryz | 1.528917375 | chr3 |
| Gbp3 | 1.516550183 | chr3 |
| Elovl6 | 1.508915256 | chr3 |
| Slc19a2 | 1.507126743 | chr1 |
| Tardbp | 1.482860803 | chr4 |
| Tcp1 | 1.48075612 | chr17 |
| 2010305A19Rik | 1.47781646 | chr4 |
| Memo1 | 1.456344955 | chr17 |
| NAP101637-1 | 1.453163332 | chr1 |
| Ltb4dh | 1.452581632 | chr4 |
| Pet112l | 1.449396 | chr3 |
| 5730437N04Rik | 1.441433694 | chr17 |
| Eif2a | 1.416705903 | chr3 |
| Hdgf | 1.399624158 | chr3 |
| Gmps | 1.397429025 | chr3 |
| Hist2h2aa1 | 1.391945976 | chr3 |
| Pla2g12a | 1.375802946 | chr3 |
| Smyd2 | 1.371691256 | chr1 |
| Yars | 1.365891258 | chr4 |
| Cd28 | 1.35274958 | chr1 |
| Vps45 | 1.351446694 | chr3 |
| Zbtb8os | 1.34686533 | chr4 |
| Bcs1l | 1.331575056 | chr1 |
| Flywch1 | 1.323786828 | chr17 |
| 2010300C02Rik | 1.311382018 | chr1 |
| Mrps21 | 1.310231174 | chr3 |
| Osgepl1 | 1.300453086 | chr1 |
| Hsdl2 | 1.294721342 | chr4 |
| Psmd1 | 1.292952129 | chr1 |
| Tnfsf8 | 1.291434432 | chr4 |

| | | |
|---|---|---|
| Slc31a1 | 1.287898901 | chr4 |
| Lzic | 1.278972525 | chr4 |
| BC033430 | 1.267329443 | chr1 |
| Plrg1 | 1.264075519 | chr3 |
| Fh1 | 1.255127262 | chr1 |
| Pars2 | 1.233471339 | chr4 |
| Nhej1 | 1.227266364 | chr1 |
| Anp32e | 1.216937037 | chr3 |
| Aldh9a1 | 1.211196454 | chr1 |
| Mrpl2 | 1.199216577 | chr17 |
| Acad9 | 1.19754006 | chr3 |
| Faf1 | 1.197447108 | chr4 |
| Ints7 | 1.191281424 | chr1 |
| Slc16a1 | 1.18913376 | chr3 |
| Eif4e2 | 1.185496513 | chr1 |
| NAP057030-1 | 1.158570801 | chr3 |
| Mtfr1 | 1.157151626 | chr3 |
| Pgd | 1.154989094 | chr4 |
| 1110001A16Rik | 1.147372313 | chr17 |
| Abcb6 | 1.144013399 | chr1 |
| Actl6a | 1.143290408 | chr3 |
| Scye1 | 1.137678283 | chr3 |
| Wdr12 | 1.136585771 | chr1 |
| Hrb | 1.096305378 | chr1 |
| Ccrn4l | 1.091495382 | chr3 |
| Ndufs2 | 1.08776477 | chr1 |
| Tmem70 | 1.070266825 | chr1 |
| Etfdh | 1.065926891 | chr3 |
| Ppil3 | 1.06141881 | chr1 |
| Mrpl37 | 1.047220889 | chr4 |
| Yipf1 | 1.021852386 | chr4 |
| H2-Ke2 | 1.010799124 | chr17 |
| Mrps9 | 0.995405992 | chr1 |
| Srgap2 | 0.988319209 | chr1 |
| Sytl1 | 0.985181245 | chr4 |
| Fbxo42 | 0.974339463 | chr4 |
| Cog6 | 0.970323004 | chr3 |
| Tcea1 | 0.961216437 | chr1 |
| Gfm1 | 0.950933676 | chr3 |
| Taf11 | 0.949353424 | chr17 |
| 2410091C18Rik | 0.927796636 | chr17 |
| Prdm16 | 0.923303827 | chr4 |
| Pde6d | 0.912526369 | chr1 |
| ENSMUST00000072587 | 0.91164531 | chr17 |
| Rbm15 | 0.911184454 | chr3 |
| Tmem112 | 0.90971648 | chr17 |

| | | |
|---|---|---|
| Ccdc28b | 0.900096772 | chr4 |
| Ttc27 | 0.893102996 | chr17 |
| Padi3 | 0.888345188 | chr4 |
| 5730449L18Rik | 0.883811622 | chr1 |
| Ak2 | 0.863776581 | chr4 |
| Glo1 | 0.853481137 | chr17 |
| Cct3 | 0.852956199 | chr3 |
| Mecr | 0.852085966 | chr4 |
| Tmem9 | 0.852057111 | chr1 |
| Mrpl9 | 0.851435977 | chr3 |
| Ppa2 | 0.847645547 | chr3 |
| Acbd6 | 0.84561378 | chr1 |
| 2210012G02Rik | 0.84505558 | chr4 |
| Rars2 | 0.825172494 | chr4 |
| NAP030020-1 | 0.821993339 | chr1 |
| Mllt11 | 0.820903993 | chr3 |
| Kcnab2 | 0.815438618 | chr4 |
| Orc2l | 0.810107426 | chr1 |
| BC057079 | 0.807194133 | chr4 |
| Trfp | 0.799127628 | chr17 |
| Selenbp1 | 0.786038353 | chr3 |
| Aco1 | 0.781915807 | chr4 |
| Zmpste24 | 0.77370657 | chr4 |
| Atic | 0.766289702 | chr1 |
| Parp1 | 0.759766863 | chr1 |
| Adss | 0.752199922 | chr1 |
| Clstn1 | 0.751803203 | chr4 |
| Mrto4 | 0.748655852 | chr4 |
| Capn10 | 0.748275987 | chr1 |
| Ppp1r8 | 0.746747419 | chr4 |
| Rpl7l1 | 0.743224504 | chr17 |
| Bag1 | 0.735824377 | chr4 |
| Smarcal1 | 0.721732306 | chr1 |
| Nek7 | 0.71765617 | chr1 |
| Rbm8a | 0.715610741 | chr3 |
| Ppie | 0.713850385 | chr4 |
| Zfp281 | 0.711264428 | chr1 |
| Rgs3 | 0.710666458 | chr4 |
| Cdc26 | 0.710076566 | chr4 |
| 9430016H08Rik | 0.707047424 | chr1 |
| Hadh | 0.704358592 | chr3 |
| AI314976 | 0.692713074 | chr17 |
| S100pbp | 0.683165426 | chr4 |
| Exosc10 | 0.668949934 | chr4 |
| Rabggtb | 0.668696636 | chr3 |
| Faah | 0.643212149 | chr4 |

| | | |
|---|---|---|
| D1Ertd161e | 0.618572452 | chr1 |
| Brp44 | 0.607742877 | chr1 |
| Wdr8 | 0.607303542 | chr4 |
| Txndc12 | 0.604963595 | chr4 |
| Nit1 | 0.586282097 | chr1 |
| Eprs | 0.581888713 | chr1 |
| Pigq | 0.551024565 | chr17 |
| Xrcc5 | 0.546956949 | chr1 |
| Ddx20 | 0.540218758 | chr3 |
| Rap1a | 0.539103472 | chr3 |
| Rbbp5 | 0.538185706 | chr1 |
| Smu1 | 0.518882345 | chr4 |
| Mapkapk2 | 0.495504096 | chr1 |
| Cd247 | 0.493062754 | chr1 |
| Tada1l | 0.491242779 | chr1 |
| Chd1l | 0.482483817 | chr3 |
| AU014645 | 0.473826698 | chr4 |
| Smap1l | 0.466808154 | chr4 |
| Zfp364 | 0.461274849 | chr3 |
| Abcf1 | 0.4563175 | chr17 |
| D4Ertd22e | 0.456256194 | chr4 |
| Bat1a | 0.449064266 | chr17 |
| Hnrpr | 0.435374509 | chr4 |
| Icmt | 0.433146073 | chr4 |
| Rfwd2 | 0.429595333 | chr1 |
| Prkcz | 0.417490604 | chr4 |
| BC038286 | 0.399454405 | chr1 |
| Creb3 | 0.376334563 | chr4 |
| Pygo2 | 0.289222966 | chr3 |
| Pdcd2 | 0.286349215 | chr17 |
| Acp6 | 0.270126298 | chr3 |
| Tmem50a | 0.269932693 | chr4 |
| Cdc42 | 0.236560869 | chr4 |
| Ttc7 | 0.228250928 | chr17 |
| Casp8 | -0.192562059 | chr1 |
| Dph2 | -0.275886818 | chr4 |
| Mib2 | -0.292705115 | chr4 |
| Tesk1 | -0.292851062 | chr4 |
| A630001G21Rik | -0.372718136 | chr1 |
| Dph5 | -0.394129282 | chr3 |
| Ccnl2 | -0.41823745 | chr4 |
| Rassf5 | -0.48735846 | chr1 |
| 4632411B12Rik | -0.497362119 | chr1 |
| AI447904 | -0.591215652 | chr1 |
| Inpp5d | -0.619772556 | chr1 |
| Ramp1 | -0.63199778 | chr1 |

| | | |
|---|---|---|
| Lrrfip1 | -0.648905601 | chr1 |
| TC1637729 | -0.660540617 | chr4 |
| Btg2 | -0.661959911 | chr1 |
| TC1640144 | -0.677613354 | chr4 |
| Akap8l | -0.683873381 | chr17 |
| Slc37a1 | -0.698201075 | chr17 |
| 1200015A19Rik | -0.732130077 | chr4 |
| H2-Ke6 | -0.761443822 | chr17 |
| Padi2 | -0.765941107 | chr4 |
| Dock10 | -0.805159573 | chr1 |
| AV381417 | -0.808697187 | chr4 |
| Zcchc7 | -0.842468091 | chr4 |
| Tmem8 | -0.896739885 | chr17 |
| Cnr2 | -0.90666299 | chr4 |
| Itfg3 | -0.917687538 | chr17 |
| 2410166I05Rik | -0.919821358 | chr4 |
| Fcgr2b | -0.952852469 | chr1 |
| Hs6st1 | -0.968621217 | chr1 |
| Dvl1 | -0.995140771 | chr4 |
| Ski | -1.000372028 | chr4 |
| Txnip | -1.009864819 | chr3 |
| AK046358 | -1.011429467 | chr3 |
| Myo1f | -1.0397672 | chr17 |
| Fcrla | -1.054792194 | chr1 |
| Gstm1 | -1.126172363 | chr3 |
| Pkd1 | -1.169565817 | chr17 |
| Atp1b1 | -1.185426996 | chr1 |
| NAP034624-1 | -1.188207021 | chr4 |
| NAP000727-001 | -1.196268671 | chr4 |
| Pax5 | -1.273085056 | chr4 |
| 2010109N14Rik | -1.28631861 | chr3 |
| H2-Q9 | -1.292094187 | chr17 |
| Zyg11b | -1.29430565 | chr4 |
| H2-DMb2 | -1.317435183 | chr17 |
| Ccnl1 | -1.320124802 | chr3 |
| AK054043 | -1.32025935 | chr4 |
| Bcar3 | -1.338729163 | chr3 |
| Ankrd12 | -1.371177463 | chr17 |
| BC028528 | -1.395782979 | chr3 |
| Dnajb10 | -1.402710587 | chr1 |
| LOC381508 | -1.424692325 | chr1 |
| Macf1 | -1.43324114 | chr4 |
| Akap2 | -1.451189095 | chr4 |
| Agrn | -1.453459948 | chr4 |
| AK032599 | -1.525196425 | chr4 |
| Cyb561d1 | -1.542460887 | chr3 |

| | | |
|---|---|---|
| F830028O17Rik | -1.622881376 | chr17 |
| Lst1 | -1.744206943 | chr17 |
| BC034637 | -1.77804507 | chr17 |
| Il18r1 | -1.867089001 | chr1 |
| Il6ra | -1.873602029 | chr3 |
| A630081D01Rik | -1.915717123 | chr1 |
| Tdrd7 | -1.953154744 | chr4 |
| ENSMUST00000097661 | -2.001638391 | chr1 |
| H2-Ob | -2.262553305 | chr17 |
| F11r | -2.284051899 | chr1 |
| Fcgr3 | -2.341872967 | chr1 |
| Pag1 | -2.419278193 | chr3 |
| Fgd2 | -2.451455852 | chr17 |
| Kif1b | -2.539825505 | chr4 |
| BC013561 | -2.6033163 | chr1 |
| Fasl | -3.119103833 | chr1 |
| Fgr | -3.206483853 | chr4 |
| Tmem51 | -3.557687227 | chr4 |
| Ppap2b | -3.569614261 | chr4 |
| Ppfia4 | -3.858851746 | chr1 |
| Sdpr | -5.509137099 | chr1 |
| 5430435G22Rik | -7.497497924 | chr1 |
